# Inherited Nitrogen Distribution Control in Covalent Organic Framework Cathodes for Efficient Electrochemical Lithium Recovery via Capacitive Deionization

**DOI:** 10.1002/advs.202417140

**Published:** 2025-04-24

**Authors:** Rajesh Dhanushkotti, Abdul Khayum Mohammed, Kayaramkodath Chandran Ranjeesh, Hema Mylnahalli Krishnegowda, Najat Maher Aldaqqa, Dinesh Shetty

**Affiliations:** ^1^ Department of Chemistry Khalifa University PO Box 127788 Abu Dhabi UAE; ^2^ Department of Studies in Physics, Manasagangotri University of Mysore Mysuru Karnataka 570 006 India; ^3^ Center for Catalysis & Separations (CeCaS) Khalifa University P.O. Box 127788 Abu Dhabi UAE

**Keywords:** brine management, capacitive deionization, circular economy, covalent organic frameworks, lithium extraction

## Abstract

The economic recovery of lithium from brine generated by desalination plants presents a promising pathway toward achieving a sustainable water desalination economy. Selectively recovering Li^+^ ions from brine is challenging due to the presence of other dominant ions. While electrochemical separation techniques, such as hybrid capacitive deionization (HCDI), offer several advantages, success largely depends on developing suitable cathodes currently limited to inorganic materials with notable constraints. Herein, the potential of controlling heteroatom distribution within 2D covalent organic frameworks (2D‐COFs) is explored for electrochemical lithium recovery. This marks the first exploration of COF cathodes for lithium extraction via HCDI. By carefully modulating the density of heteroatoms within the framework backbone, this study aims to understand their critical role better and achieve efficient cathode materials. Notably, Tta‐Dfp, the representative COF, demonstrates a lithium recovery rate of 15.7 mg g⁻¹ at 1.4 V, with a Li‐ion concentration of 300 mg L⁻¹, and exhibits ∼80% selectivity for lithium extraction. At the same time, the device achieves 97.7% capacitance retention after 500 charge‐discharge cycles. Through controlled COFs, density functional theory (DFT) analysis, and post‐electrode characterizations, we elucidate the pivotal role of nitrogen distribution in lithium recovery.

## Graphical abstract

This study presents a rational design of hetero‐atom distribution within 2D‐COFs for selective electrochemical lithium extraction through capacitive deionization. The COF's high surface area and charge density facilitate effective lithium‐ion capture, offering a scalable and sustainable approach for lithium recovery from complex brine solutions.

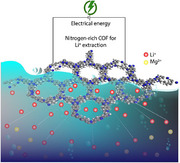

## 1 Introduction

Lithium (Li) is a vital commodity, from energy security to carbon footprint and daily life to industrial growth. The expected increase in global demand (estimated to reach $221 billion by 2024) for electric vehicles soon demands lithium‐ion batteries (LIBs).^[^ ^1^ ^]^ While global lithium production is almost constant, and the global rate of lithium recycling is only <1%. Currently, land‐based mining is the main Li‐source (>99%), facing sustainability challenges given that high‐grade mineral ore deposits are declining.^[^ ^2^ ^]^ Furthermore, mining processes are very energy and water‐intensive, and mining wastes can create lasting environmental damage. This reality has resulted in a paradigm shift toward resource recovery from waste streams.^[^ ^3^, ^4^ ^]^

Seawater and its desalination brine have recently received tremendous academic and industrial attention for their abundant resources. The large volumes of concentrated brine generated by the seawater desalination plants are estimated to be 124.5 million m^3^ day^−1^, which is generally treated as waste,^[^ ^5^, ^6^, ^7^ ^]^ is a great source of Li (element concentration is in the range of 220–3800 mg L^−1^, which after partial evaporation increases to 300–5000 ppm) that can be recovered, potentially leading to economic benefits and reduced waste disposal. Moreover, extraction of Li from concentrated brine is estimated to cost 30% to 50% less than that from mined ores.^[^ ^8^ ^]^ The economic recovery of Li from the brine concentrate would greatly support the goal of a sustainable economy for water desalination because of the reduction in the water production cost by the revenue from the recovered mineral and the environmental problems associated with brine disposal.^[^ ^9^, ^10^ ^]^ However, the selective recovery of Li^+^ ions from brine is challenging because of the presence of other dominant ions, such as sodium (Na^+^), potassium (K^+^), calcium (Ca^2+^), and magnesium (Mg^2+^), at higher concentrations.^[^ ^11^ ^]^ Traditional chemical precipitation and solvent extraction methods generate large volumes of sludge, making them less environmentally friendly. Among the various research approaches explored for recovering lithium from seawater and its brine, adsorption and electrochemical separation methods stand out due to their higher elemental selectivity, low energy requirements, and chemical modification and operation flexibility, including potential integration into a unified system.^[^ ^12^, ^13^, ^14^ ^]^ However, the search for optimal adsorbents with high lithium uptake, chemical stability, selectivity, and the development of novel electrochemical methods is still in its early stages.^[^ ^15^ ^]^

Meanwhile, hybrid capacitive deionization (HCDI)—a battery‐like process in which ions are adsorbed onto an electrode surface under an applied electric field—has emerged as a promising method for lithium extraction.^[^ ^16^, ^17^ ^]^ This approach offers several advantages, including lower energy consumption, rapid kinetics, and selectivity. Inorganic materials such as MnO₂, LiFePO₄//Ag, λ‐MnO₂//Ag, and LMO//Zn have been well‐studied as electrodes for lithium recovery from brine.^[^ ^18^, ^19^ ^]^ However, these materials face challenges such as toxicity, structural stability, higher energy consumption, and limited functional tunability, which hinder their ability to achieve the desired lithium extraction efficiency and selectivity. In contrast, covalent organic frameworks (COFs)—a class of crystalline polymer materials—offer several advantages, including porosity, functional tunability, and high surface area.^[^ ^20^, ^21^, ^22^, ^23^ ^]^ However, there are currently no reports exploring COF‐based electrode materials for lithium extraction via HCDI.

Recognizing this opportunity, we have rationally designed imine‐linked nitrogen‐rich 2D‐COFs as cathode materials for effective lithium recovery from brine solutions in an HCDI system. Among the tested COFs, Tta‐Dfp was particularly effective, achieving a lithium uptake of 15.7 mg g⁻¹ at 1.4 V with a Li‐ion concentration of 300 mg L⁻¹ and exhibiting ≈80% selectivity for lithium extraction. This effectiveness is likely due to pyridine, imine, and triazine nitrogen atoms, which facilitate reversible lithium interactions with the COF under applied potential. We systematically studied the influence of the nitrogen atoms in the COFs on lithium extraction by varying their abundance. Detailed density functional theory (DFT) studies supported our findings and elucidated the interactions between the COF backbone and Li⁺ ions. This rational molecular engineering of COFs for efficient lithium extraction via HCDI could pave the way for addressing the challenges associated with existing inorganic electrode materials.

## 2 Result and Discussion

### 2.1 Synthesis and Characterizations of COFs

**Figure 1:**
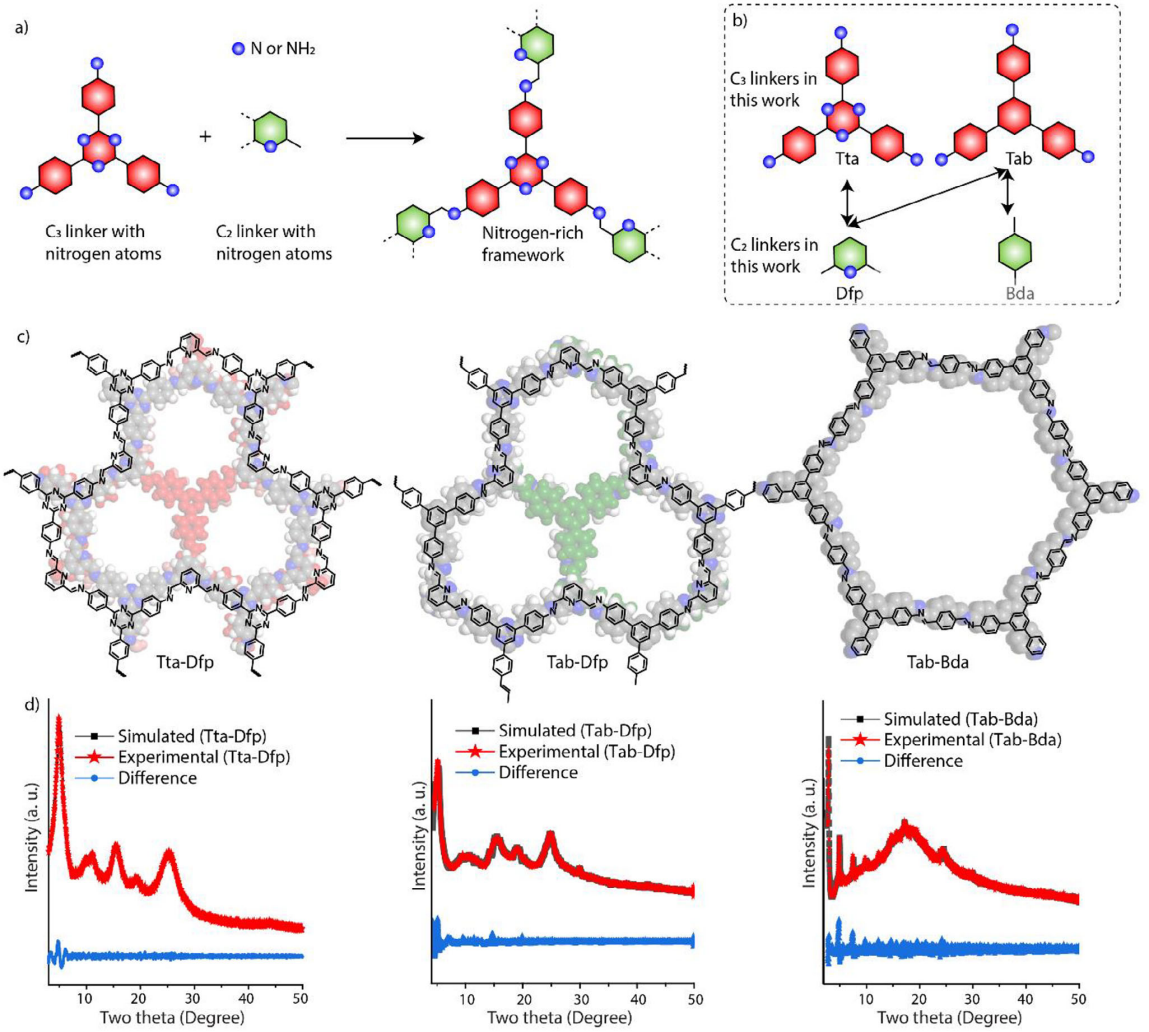
a,b) The diagrammatic representation of inherited nitrogen atoms distribution control within the framework by rational building block selection. c) The ChemDraw image of Tta‐Dfp, Tab‐Dfb, and Tab‐Bda. d) The experimental and calculated PXRD profiles of Tta‐Dfp, Tab‐Dfb, and Tab‐Bda.

Design and synthesis: Our strategy to enhance the performance of the cathode material for lithium extraction in the HCDI setup involves incorporating and controlling nitrogen content within the framework. To demonstrate the critical role of nitrogen in COFs, we synthesized three COFs (Tta‐Dfp, Tab‐Dfp, and Tab‐Bda) with a controlled number of nitrogen atoms(**Figure** **1**a–c).^[^ ^24^ ^]^ Importantly, all COFs were synthesized at identical conditions to minimize the influence of external variables in CDI performance (Section S2, Supporting Information). The COFs were synthesized using the Schiff base condensation reaction of amine and aldehyde organic building blocks in 2 mL of a 1, 4‐dioxane/mesitylene mixture (1:1 v/v) with a catalytic volume of acetic acid (50 µL, 6 m) in a sealed pressure tube. The reaction mixture was heated at 120 °C for 5 days, and then the precipitate was collected and washed with N, N'‐dimethylacetamide (DMA), water, and acetone. Notably, Tta‐Dfp features a dense distribution of nitrogen within the framework, contributed by pyridine, imine, and triazine nitrogen atoms. In contrast, Tab‐Dfp and Tab‐Bda contain either pyridine/imine or imine nitrogen atoms.

Structural analysis: The powder X‐ray diffraction (PXRD) profiles of the synthesized COFs closely match the corresponding simulated structures and are consistent with the reported literature^[^ ^23^, ^25^ ^]^ (Figure 1c). The patterns reveal two distinct peaks: the peak at 2*θ* = 5.0° corresponds to the (110) plane of the well‐ordered lattice, while the broad peak centered at 2*θ* = 25° is associated with reflections from the (003) plane. Notably, the layer‐stacked structures with an ABC sequence exhibited the best alignment between the experimental and simulated PXRD patterns (Figure S1, Supporting Information), with R_wp_ and R_p_ values of 2.60% and 1.98% for Tta‐Dfp, 3.11% and 1.89% for Tab‐Dfp, and 5.35% and 3.57% for Tab‐Bda, respectively.

Analysis of the chemical environment of COFs: The Fourier‐transform infrared (FT‐IR) spectra provide evidence of imine (C═N) stretching vibrations at ≈1690 cm⁻¹ in the Tta‐Dfp, Tab‐Dfp, and Tab‐Bda COFs. A comparison of the FT‐IR spectra of the pure monomers reveals that the stretching bands for the aldehyde (1720–1735 cm⁻¹) and amine groups (3300–3400 cm⁻¹) are significantly weaker in COFs, which indicates a successful imine condensation reaction (Figures S2–S4, Supporting Information). It is important to note that the polymerization process during COF formation may not have been fully completed, resulting in a trace peak at 1720–1735 cm⁻¹ in the spectra of Tta‐Dfp, Tab‐Dfp, and Tab‐Bda. This peak is likely due to unreacted peripheral end functional groups that persist during the 2D growth of the COFs.^[^ ^24^ ^]^ The formation of COFs was subsequently confirmed through ^13^Carbon cross‐polarization magic‐angle spinning nuclear magnetic resonance (CP‐MAS NMR) spectroscopy (Figure S5, Supporting Information). The NMR spectra of Tta‐Dfp revealed a distinctive peak at ≈157 ppm, corresponding to the imine carbon (─C═N), which endorses the imine‐linked COF formation. We observed additional peaks for Tta‐Dfp at 114–137, 154, and 168 ppm corresponding to the carbon atoms within the phenyl, triazine, and pyridine moieties, respectively. The peaks observed in the Tab‐Dfp NMR spectrum at ≈154 ppm correspond to imine carbon (─C═N), whereas ≈168 ppm and 138–112 ppm are associated with the carbon atoms in the pyridine and phenyl rings, respectively. Similarly, Tab‐Bda exhibits a distinctive peak at ≈156 ppm, corresponding to the imine carbon (─C═N). The remaining aromatic carbons of the Tab‐Bda structure resulted in peaks at 137–115 ppm range.^[^ ^26^, ^27^ ^]^

The X‐ray photoelectron spectroscopy (XPS) survey spectra of Tta‐Dfp, Tab‐Dfp, and Tab‐Bda displayed peaks at binding energies (BE) of ≈284 and 396.9 eV corresponding to C 1s, and N 1s, respectively, in the survey spectra^[^ ^23^, ^31^ ^]^ (Figure S6 Supporting Information). The stronger N 1s peak at 396.9 eV in Tta‐Dfp indicates a higher atomic nitrogen ratio than Tab‐Dfp and Tab‐Bda. The C 1s XPS spectra for Tta‐Dfp were deconvoluted, revealing the presence of aromatic C═C bonds, C═N bonds (pyridine), C═N bonds (imine linkage), C═N (triazine), and a carbon satellite. These features were identified at binding energy values of 284.5, 286.4, 288.1, and 290.3 eV, respectively. Notably, Tta‐Dfp also exhibited a C═N (triazine) peak at 285.7 eV, which was absent in Tab‐Dfp. Furthermore, the N 1s XPS spectra of Tta‐Dfp showed three peaks at binding energies of 398.8, 399.6, and 401.2 eV, corresponding to C═N (triazine), C═N (imine), C═N (pyridine), and a satellite peak, respectively. In contrast, Tab‐Dfp does not contain the triazine moiety, while Tab‐Bda lacks both C═N (triazine) and C═N (pyridine) bonds, featuring only C═N (imine) bonds.

Morphological analysis: All COFs underwent SEM analysis and. revealed a sheet‐like morphology for Tta‐Dfp (Figure S7, Supporting Information). Meanwhile, Tab‐Dfp exhibited an agglomerated particulate morphology. Notably, Tab‐Bda demonstrated a sphere‐like particulate structure, with a size ranging from 0.5 to 1.5 µm. Energy‐dispersive X‐ray spectroscopy (EDS) elemental dot mapping confirms the presence of both nitrogen (N) and carbon (C) within the framework structure. Specifically, the EDS mapping for Tta‐Dfp demonstrates a uniform distribution of nitrogen and carbon throughout the framework (Figure S7, Supporting Information). Additionally, the TEM images show the characteristic nano‐level morphology of each COF: Both Tta‐Dfp and Tab‐Dfp feature the lamellar‐like structures. Meanwhile, Tab‐Bda exhibits a sphere‐like morphology where the spheres (with a spherical diameter of ≈641 nm) are composed of stacked layers (Figure S8, Supporting Information).

Porosity analysis: In addition, the N_2_ adsorption experiments on Tta‐Dfp, Tab‐Dfp, and Tab‐Bda revealed the microporous nature of the material, with a Brunauer‐Emmett‐Teller (BET) surface area of 417, 369, and 28 m^2^ g^−1^ respectively. In addition, Tta‐Dfp, Tab‐Dfp, and Tab‐Bda displayed a maximum pore size distribution of ≈1.45, ≈1.40, and ≈2.5 nm, respectively (Figure S9, Supporting Information). These values align with the simulated COF structures.^[^ ^28^, ^29^, ^30^ ^]^ Both Tta‐Dfp and Tab‐Dfp exhibit similar pore size distributions, while Tab‐Bda demonstrates a larger pore size due to its AA stacking framework, as opposed to the ABC stacking in Tta‐Dfp and Tab‐Dfp, which results in smaller pores (Figure S10, Supporting Information). Furthermore, the framework of Tab‐Bda incorporates only imine‐type nitrogen. In this regard, a smaller pore size imine‐based COF, Tfp‐Pda, has been synthesized to match the pore sizes of Tta‐Dfp and Tab‐Dfp, while maintaining a framework composed solely of imine nitrogen. Tfp‐Pda was synthesized from 1, 3, 5‐triformylbenzene (Tfb) and p‐phenyldiamine (Pda) through a Schiff base condensation reaction under similar synthetic conditions (Figure S11a, Supporting Information). PXRD analysis confirmed the crystalline nature of Tfp‐Pda and its alignment with the theoretical AA‐stacking model (Figure S11b,c, Supporting Information). The FT‐IR profiles indicated the formation of imine (─C═N) bonds (Figure S11d, Supporting Information). The XPS analysis showed sp^2^ carbon and imine nitrogen in the framework (Figure S11e, Supporting Information). Notably, Tfp‐Pda has a comparable pore size (≈1.5 nm) to that of Tta‐Dfp and Tab‐Dfp and also contains only imine nitrogen in its structure (Figure S11f,g, Supporting Information). SEM and TEM analyses revealed a spherical morphology, similar to that of Tab‐Bda, indicating Tfp‐Pda as a smaller pore size analogue to Tab‐Bda (Figure S11h,i, Supporting Information).

### 2.2 Electrochemical Properties

**Figure 2:**
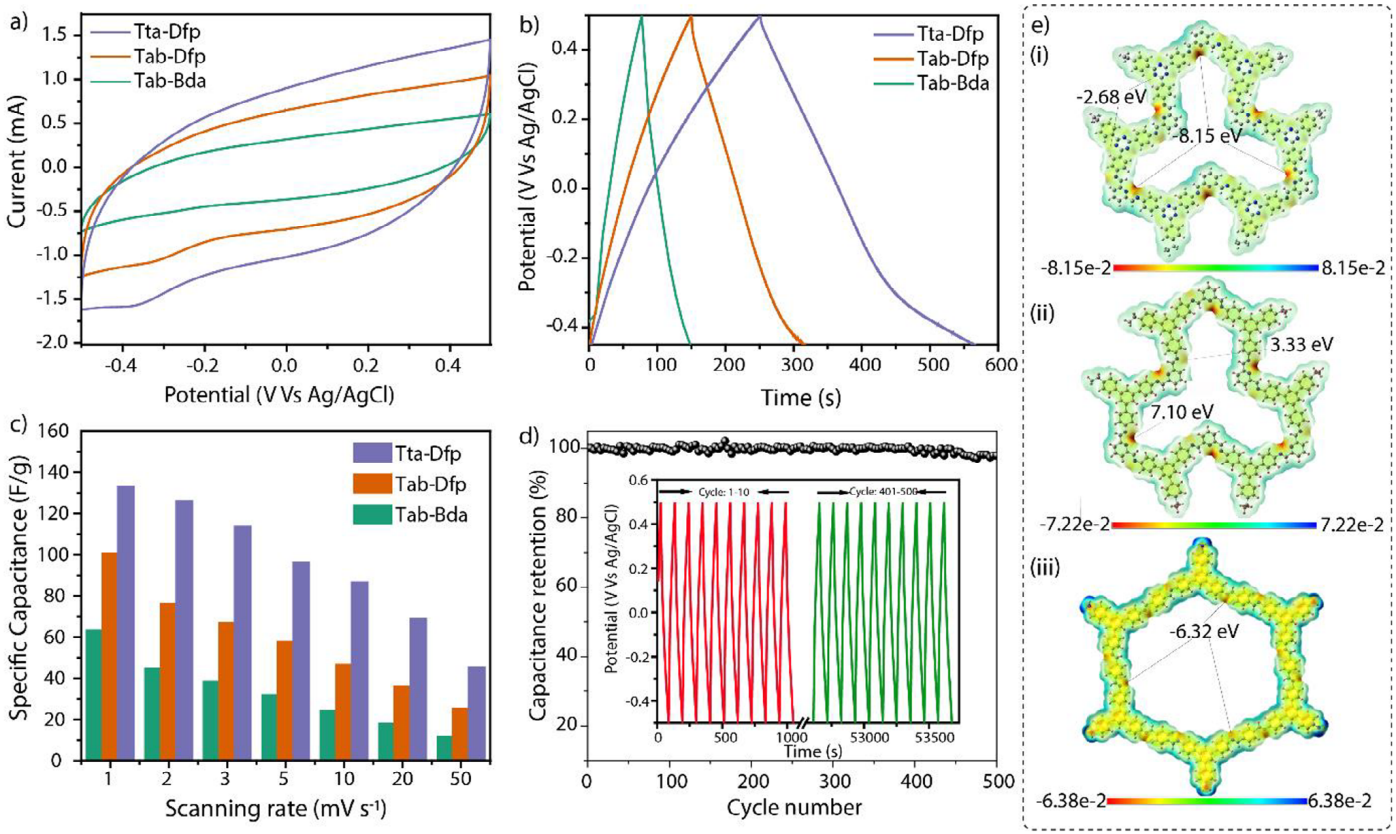
a) CV curves, b) GCD plots, and c) Specific capacitance curves for COFs tested in this study. d) Cyclic stability (500 cycles) of the Tta‐Dfp COF. e) Selected area ESP mapping of i) Tta‐Dfp, ii) Tab‐Dfb, and iii) Tab‐Bda showing the negative charge polarization (red color) toward triazine, pyridine, and imine nitrogen cores.

Three‐electrode analysis: To understand COF interactions with Li^+^ ions, we have characterized the electrode properties in a three‐electrode system using COF as a working electrode, Ag/AgCl as a reference electrode, and Platinum as a counter electrode in 1 M LiCl electrolyte. The cyclic voltammetry (CV) analysis shows an electric double‐layer capacitive (EDLC) nature with a specific capacitance of 132, 100, and 63.11 F g^−1^ at 1 mV s^−1^ for Tta‐Dfp, Tab‐Dfp, and Tab‐Bda, respectively (**Figure** **2**a,c). The EDLC nature indicates the electrostatic interaction of Li^+^ ions with COFs, whereas the difference in specific capacitance indicates the extent of these interactions. The better EDLC performance of Tta‐Dfp is validated because of improved accessibility and stronger interactions of densely distributed nitrogen atoms with Li⁺ ions. The high number of electronegative nitrogen atoms within imine, pyridine, and triazine functional cores and their uniform distribution within Tta‐Dfp contribute to its highest specific capacitance among the three COFs. Notably, there is a direct correlation between observed specific capacitance against nitrogen atom density within COFs.^[^ ^32^ ^]^ Under an electric field, nitrogen atoms are getting polarized, which enhances their ability to donate electrons and subsequent effective interaction with Li⁺ ions. The increased electrostatic interactions drive the improved electrosorption process, thereby higher capacitance observed in Tta‐Dfp. We have found minimal impact on the CV curve shapes of COFs within scanning rates ranging from 1 to 50 mV s^−1^ (Figures S12a,b and S13a,b, Supporting Information), indicating well‐maintained capacitive performance even at higher scan rates.

The galvanostatic charge‐discharge (GCD) curves (Figure. 2b) at a current density of 0.1 A g^−1^ for COFs confirm the reversibility of the electrode reactions with an EDLC behavior (Figures S12c,d and S13c,d, Supporting Information). The stronger electrostatic interactions between the nitrogen atoms in Tta‐Dfp and the Li⁺ under an applied electric potential are likely to enhance ion adsorption and desorption kinetics, leading to well‐defined GCD curves with minimal voltage drop and higher energy retention. In contrast, COFs with lower nitrogen content exhibit weaker interactions resulting in less efficient ion diffusion and slightly lower ion storage efficiency, as reflected in their GCD profiles.

The EIS plots were conducted to compare the efficiency of Li^+^ ion and electron transport in COF electrodes (Figure S14, Supporting Information). The Tta‐Dfp electrode exhibits a significantly lower charge transfer resistance (Rct: 3.3 Ω) than other COF electrodes (Table S1, Supporting Information). Tab‐Dfp and Tab‐Bda recorded higher Rct values of 7.4 Ω and 15 Ω, respectively. Notably, the lower Rct of Tta‐Dfp indicates faster ion/electron kinetics than those of Tab‐Dfp and Tab‐Bda, which enhances the electrosorption capacity of Tta‐Dfp. The molecular differences among these COFs, particularly the nitrogen density in the frameworks, directly impact their charge‐discharge behaviors. The nitrogen sites become more polarized under electric potential, facilitating effective interaction with Li^+^ ions, thus lowering the resistance at the electrode‐electrolyte interfaces. Conversely, the reduced number of nitrogen sites in Tab‐Dfp and Tab‐Bda increases this interface resistance, thereby decreasing ion kinetics and electrosorption capacity. The cyclic stability analysis demonstrated that the Tta‐Dfp maintains 98% of its initial capacitance even after 500 cycles, highlighting the electrochemical stability of the material (Figure 2d). Specifically, the capacitance variation from cycle 1 to 350 remains below 1% of the initial capacitance. After 400 cycles, the capacitance retention slightly decreases to 98.8%. Notably, this level of capacitance retention was consistently maintained throughout the entire charge‐discharge cycling analysis.^[^ ^32^, ^33^ ^]^ It is noteworthy to mention that cyclic stability is tested only for Tta‐Dfp because of its superior capacitance behavior.

ESP mapping: To further analyze the local polarization and charge separation behavior of the synthesized COFs, the electrostatic potential (ESP) mapping calculations were performed (Figure 2e). The ESP results indicate that the regions of maximum negative charge (showed in red color) are predominantly located within triazine, pyridine, and imine functional pockets. Notably, Tta‐Dfp exhibits the highest negative charge density value (−8.15e^−2^ eV) followed by Tab‐Dfp (−7.22e^−2^ eV) and Tab‐Bda (−6.33e^−2^ eV). The ESP trend clearly indicates higher nitrogen atom distribution within Tta‐Dfp, leading to stronger interactions with Li⁺ ions during electrosorption. Conversely, COFs with lower ESP values exhibit reduced ion adsorption capacity and lower specific capacitance. These observations align with the enhanced electrochemical performance observed in cyclic voltammetry and galvanostatic charge‐discharge (GCD) tests for Tta‐Dfp compared to the other COFs.

**Figure 3:**
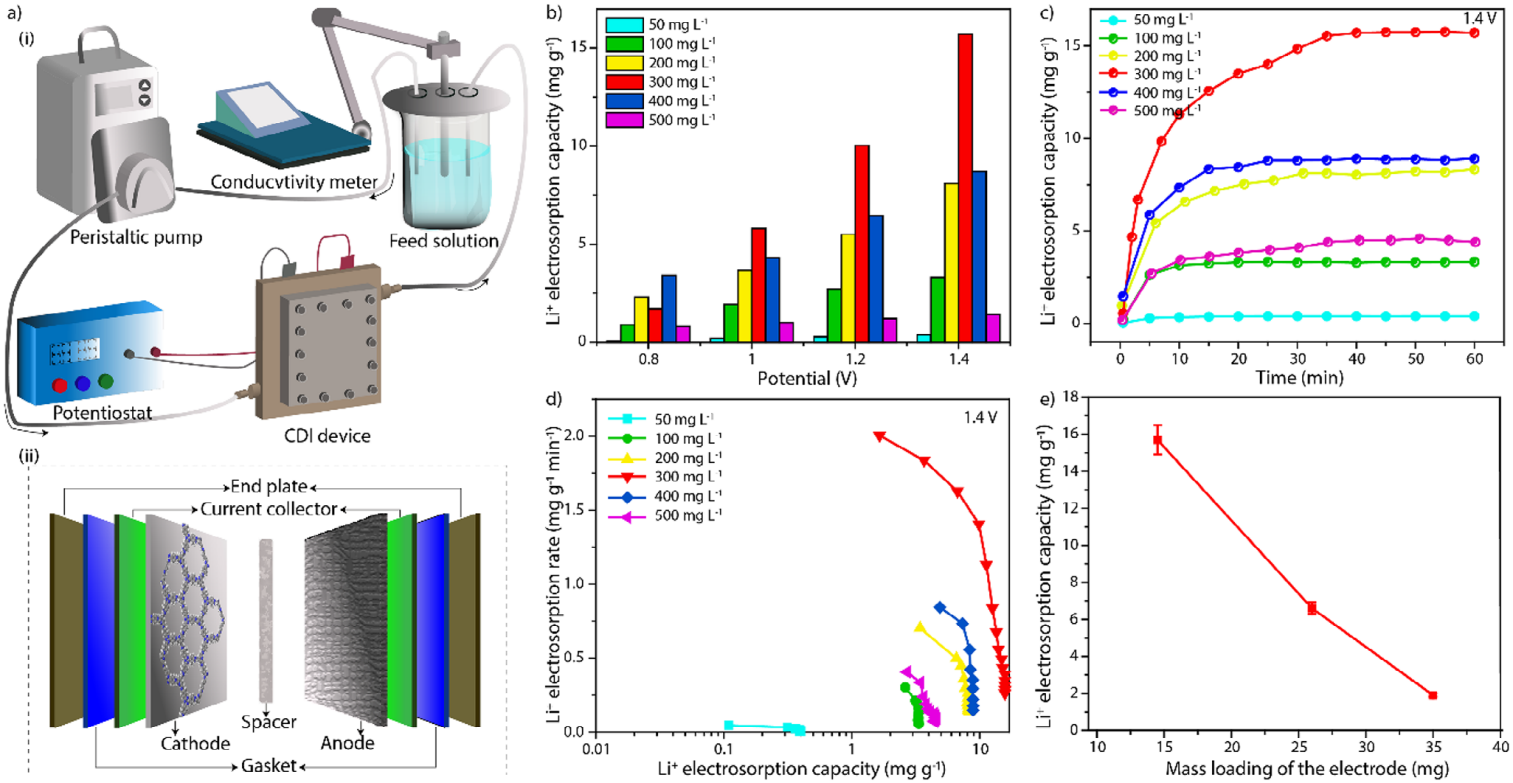
a) Schematic diagram of the HCDI setup while Tta‐Dfp was used as a cathode material. b) Electrosorption capacity of the Tta‐Dfp electrode at various Li^+^ ion concentrations. c) Electrosorption capacity of Tta‐Dfp electrode in different Li^+^ ion concentrations at 1.4 V. d) corresponding Kim–Yoon plot. e) Li ions electrosoprtion results against mass loading of Tta‐Dfp; concentration 300 mg L^−1^ at 1.4 V.

The HCDI device testing: The exceptional electrochemical behavior including excellent EDLC, high specific capacitance, and impressive cyclic stability exhibited by Tta‐Dfp prompted us to further investigate it as an electrode material for lithium extraction via HCDI (**Figure** **3**). Figure 3a provides a detailed schematic of the HCDI system: it consists of two electrodes each with a geometrical area of 16 cm^−2^, Tta‐Dfp cathode, and activated carbon (AC) anode. The Tta‐Dfp//AC asymmetric HCDI device was tested for Li^+^ ions extraction from various LiCl concentration solutions (50 to 500 mg L^−1^). The lithium extraction capacity was linearly increased along with the LiCl concentration up to 300 mg L^−1^ and then started to decrease at higher concentrations (Figure 3b,c; Figures S15–S17, Supporting Information), which could be due to the following reasons: 1) at lower concentrations, the linear increment in uptake capacity is credited to the unavailability of enough Li^+^ ions near the surface of the electrode and 2) when the concentration of electrolyte increases beyond the optimum concentration, Li^+^ ions largely adsorb on the outer surface of the electrode and block lithium diffusion into deeper pockets of the electrode although the micropore channels created by the ABC stacking of layers within Tta‐Dfp are suitable for lithium intercalation.

The observed shift toward the top right within the Kim–Yoon plot confirms the rate kinetics and electrosorption capacity of Tta‐Dfp. The findings indicate the relation between applied voltage and rate kinetics with the optimal performance observed at ≈1.4 V. At elevated voltages, the intensified electric field accelerates ion transport and adsorption, resulting in superior rate kinetics (Figure 3d). Moreover, Li⁺ electrosorption rate exhibits a characteristic pattern along with the Li⁺ concentrations within a solution (Figures S15–S17, Supporting Information): the rate was increased (from 0.04 to 0.70 mg g⁻¹ min⁻¹) at lower concentrations (50 to 200 mg L^−1^), which is attributed to the higher availability of Li⁺ ions at the active sites in electrode, which creates a steeper concentration gradient. This gradient acts as a driving force, enhancing the mass transfer of Li⁺ ions to the electrode surface. At 300 mg L^−1^, the electrosorption rate peaks at 2.04 mg g⁻¹ min⁻¹ (Figure S16d, Supporting Information). This concentration represents the most efficient condition for Li⁺ adsorption, where the balance between the number of available Li⁺ ions and the electrode's adsorption capacity is optimal. The strong concentration gradient provides an effective driving force for mass transfer while the electrode surface still has enough active sites for Li⁺ adsorption. At higher concentrations (400 and 500 mg L^−1^), the electrosorption rates decline to 0.84 and 0.57 mg g⁻¹ min⁻¹, respectively (Figure S17c,d, Supporting Information). This decrease is likely due to the saturation of adsorption sites on the electrode. The saturation of active sites limits the electrode's capacity to adsorb more ions, even though more Li ions are in the solution.

Because of electron‐donating properties, the abundant nitrogen atoms within Tta‐Dfp play a pivotal role in enhancing the electrosorption by attributing a higher affinity for Li⁺ ions.^[^ ^34^ ^]^ Furthermore, we also investigated the impact of active material mass loading in electrodes (Tta‐Dfp mass was varied from 14.5 to 35 mg) under various LiCl concentrations and applied potentials (0.8 to 1.4 V). Notably, 14.5 mg Tta‐Dfp loaded electrode showed superior Li⁺ ions adsorption performance compared to other electrodes (Figure 3c). The Tta‐Dfp//AC device demonstrated the highest lithium uptake capacity of 15.7 mg g^−1^ at 1.4 V for 300 mg L^−1^ LiCl concentration. This enhancement is attributed to the increased interaction between Li^+^ ions and Tta‐Dfp at higher voltages because of elevated ion migration.^[^ ^35^, ^36^ ^]^ It is important to note that the electrosorption capacity versus time indicates adsorption saturation at the applied potential: an increase from 0.8 to 1.4 V resulted in increased saturation time, rising from 5 minutes to 25 min (Figure 3d). This trend indicates that higher potentials enhance the electrosorption capacity and prolong the time needed for the system to achieve maximum ion uptake. In addition, the enhanced electric field strength within the HCDI device at elevated potentials creates a stronger attraction between Li^+^ ions and the Tta‐Dfp electrode. While this stronger field facilitates more effective ion adsorption, it can also slow ion mobility, requiring ions to overcome greater energetic barriers to access available adsorption sites, thereby leading to a more gradual approach to saturation. The electrode's efficiency is further supported by its favorable performance at a mass loading of 14.5 mg, indicating its potential for scalable applications^[^ ^37^ ^]^ (Figure 3e; Figures S18 and S19, Supporting Information). The stability of the Tta‐Dfp was investigated in 30 mg L^−1^ LiCl solution with continuous charge/discharge cycles for 1000 min, and the material showed consistent performance, indicating stability. (Figure S20, Supporting Information).

Furthermore, to ensure the graphite foil substrate interference in adsorption, a preliminary adsorption test was performed on pristine graphite foil, and we found no interactions with Li^+^ ions (Figure S21, Supporting Information). Moreover, the lithium extraction capabilities of Tab‐Dfp and Tab‐Bda have been assessed using HCDI (Figure S22, Supporting Information). Interestingly, The findings from HCDI analysis further confirm that nitrogen content significantly influences lithium recovery efficiency. In the HCDI test, Tab‐Dfp as a cathode exhibited moderate lithium recovery efficiency (4.5 mg g^−1^). Notably, Tab‐Bda showed poor lithium extraction (<0.3 mg g^−1^), owing to its lower number of active nitrogen sites. Among the three COFs, Tta‐Dfp demonstrated the highest lithium recovery capacity, attributed to its higher nitrogen content, enhancing interactions with Li⁺ ions. Overall, the Tta‐Dfp showed a notable 32% recovery of lithium from the flow electrolyte after one cycle. In contrast, the Tab‐Dfp achieved only 9.1% lithium recovery, while Tab‐Bda exhibited poor lithium recovery (<1%). Notably, Tfb‐Pda, the smaller pore size (≈1.5 nm) analog of Tab‐Bda, also showed a similar poor lithium recovery performance. Importantly, the three‐electrode analysis of Tfb‐Pda revealed a lithium capacitance of 40 F g^−1^, lower than that of all three other COFs (Figure S23, Supporting Information). Additionally, the HCDI analysis of Tfb‐Pda showed poor performance in lithium capture (<0.1 mg g^−1^) (Figure S22, Supporting Information). This suggests that the capability of lithium capture efficiency is predominantly influenced by the nitrogen content in these systems, not by the pore sizes.

The nitrogen sites are predominantly basic and can interact with Lewis acidic cations like lithium ions. When nitrogen density increases, lithium uptake is exponentially increasing in CDI. The presence of several nitrogen atoms in Tta‐Dfp aids in better contact with electrolyte, which enhances the mass transfer of lithium. The DFT‐optimized structures of Tta‐Dfp indicate an interaction of lithium with the cis‐imine‐pyridine nitrogen pocket, showing bond distances of ≈1.91 Å (with pyridine nitrogen) and 1.93 Å (with cis‐imine nitrogen) and the lowest binding energy of −83.0 kcal mol^−1^ (Figures S24a and S25a, Supporting Information). Interestingly, the interaction between lithium and the trans‐imine nitrogen exhibits a shorter bond distance (1.85 Å), possibly due to reduced steric hindrance from the trans‐imine bond, however, slightly higher binding energy (−75.8 kcal mol^−1^ (Figures S24b and S25b, Supporting Information). Meanwhile, interactions between lithium and triazine nitrogen atoms occur at bond distances of 1.89–1.92 Å, with relatively higher binding energy (−66.5 kcal mol^−1^ (Figures S24c and S25c, Supporting Information). Overall, all nitrogen atoms in the framework can interact with lithium, making Tta‐Dfp a more lithiophilic framework than Tab‐Dfp and Tab‐Bda, which contain fewer nitrogen atoms. The charge transfer and exciton analysis further highlights the influence of nitrogen atoms in electronic properties, where the HOMO orbitals are localized around pyridine and imine sites, facilitating fast electron transfer and Li⁺ stabilization, while the delocalized LUMO orbitals enable efficient charge mobility, ensuring rapid adsorption and desorption during CDI cycles (Figure S25d, Supporting Information). The quantitative charge transfer and exciton analysis (Figure S25, Supporting Information) further highlights the high electronic conductivity and efficient charge separation in Tta‐Dfp.

**Figure 4:**
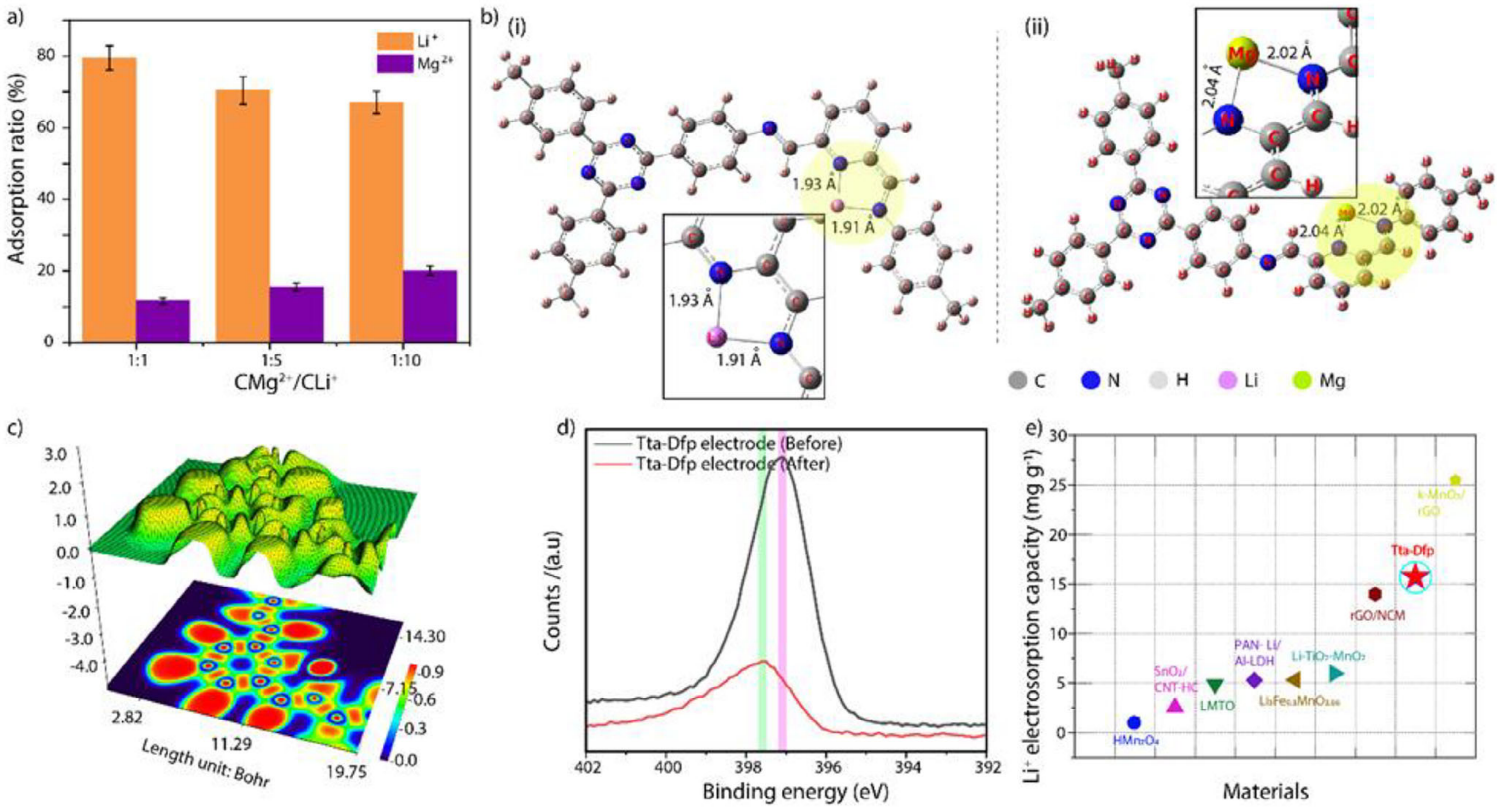
a) Electrosorption capacity of Tta‐Dfp electrodes under different molar ratios of Mg^2+^:Li^+^. b) (i) the theoretical model of a fragment of Tta‐Dfp with Li^+^ interaction; (ii) Tta‐Dfp with Mg^2+^ interaction. c) Electron Localization Function (ELF) color filled (bottom) and contour map (top) image of Tta‐Dfp after lithium incorporation d) XPS spectrum N1s of Tta‐Dfp before (black color) and after (red color) CDI test. e) Comparison of the electrosorption capacity of Li^+^ to other materials.

It is important to note that the selective extraction of Li^+^ ions in the presence of magnesium ions (Mg^2+^) is challenging because of the similar ionic radius [Mg^2+^ (72 pm) and Li^+^ ions (76 pm)] and higher positive charge associated with Mg^2+^ leading to competitive interaction with the deionization electrode. Such a challenge requires electrode materials with functional groups that can preferentially interact with Li^+^ over Mg^2+^. Interestingly, the Tta‐Dfp||AC device showed impressive selectivity of 79.6% for Li^+^ ions when we performed the experiment with C_Mg_^+^: C_Li_^+^ ratio of 1:1^[^ ^38^ ^]^(**Figure** **4**a). Notably, at higher concentrations of Mg^2+^, the selectivity toward Li^+^ is slightly decreased to 67.05% due to the abundance of Mg^2+^.

Density functional theory (DFT) calculations were performed to further investigate the selectivity, revealing that Li⁺ forms more interactions with pyridine‐imine nitrogen atoms than Mg^2^⁺ (Figure S26, Supporting Information). Specifically, bond distances between Mg^2^⁺ and the imine/pyridine nitrogen atoms were found to be longer (2.02 and 2.04 Å, respectively), with a binding energy of −83.216 kcal mol^−1^, while Li⁺ exhibited shorter bond lengths (1.91 Å for imine N–Li and 1.93 Å for pyridine N–Li) with a binding energy of −83.051 kcal mol^−1[^ ^39^, ^40^ ^]^ (Figure 4b). Despite the slightly lower binding energy for Li⁺, its smaller ionic radius and more favorable spatial arrangement allow for electrostatic interactions with the nitrogen sites. Moreover, the solvation shell around Li⁺ is typically smaller and less tightly bound than that of Mg^2^⁺, which further enhances Li⁺ ability to form closer and more direct interactions with the nitrogen sites, compensating for the lower charge density compared to Mg^2^⁺.^[^ ^39^ ^]^ The Electron Localization Function (ELF) analysis revealed that Tta‐Dfp shows higher electron localization, particularly around the nitrogen atoms, especially in the pyridine‐imine region (Figure S27, Supporting Information). This suggests that Li⁺ interacts with these nitrogen sites (Figure 4c). The observation supports that the interactions of Li⁺ are not merely driven by electrostatic forces but also by additional factors such as electron donation or polarization effects at specific sites.

Furthermore, to understand the interaction between lithium and nitrogen in Tta‐Dfp COF, ex situ X‐ray photoelectron spectroscopy (XPS) analysis was performed before and after HCDI. The N1s peak is slightly shifted to higher binding energy (397.88 eV) post‐adsorption than the pristine electrode (397.58 eV), plausibly because of Li—N interactions (Figure 4d). The electron‐rich nitrogen sites facilitate Li^+^ ion interactions to enhance the adsorption process. Notably, the performance of Tta‐Dfp was compared with reported inorganic cathodes, demonstrating its superior electrosorption capacity even at lower concentrations (Figure 4e; Table S2, Supporting Information). Notably, the PXRD analysis of Tta‐Dfp cathode in the HCDI device after the lithium extraction cycling process showed the diffraction peaks with varying intensity compared to the pristine Tta‐Dfp. The broadening of the peak intensity of the 110 planes indicates the delamination of 2D‐COF layers during lithium interactions^[^ ^23^ ^]^ (Figure S28, Supporting Information). The 2D layers of Tta‐Dfp are packed through ABC stacking, which provides only fewer π‐ π stacking between the layers and is susceptible to delamination because of Li‐intrercalation. Furthermore, the similarity of FT‐IR profiles of Tta‐Dfp cathode before and after the lithium extraction indicates the chemical stability of the material during the adsorption process (Figure S29, Supporting Information). The SEM images showed a similar morphology for both before and after electrode analysis (Figure S30, Supporting Information), indicating intact morphology of the material.

## 3 Conclusion

In conclusion, this study underscores the pivotal role of heteroatoms in enhancing the efficiency of 2D‐COFs as electrodes for electrochemical lithium recovery. The nitrogen‐rich Tta‐Dfp demonstrated a substantial lithium recovery capacity of 15.7 mg g^−1^, significantly outperforming Tab‐Dfp (4.5 mg g^−1^) and Tab‐Bda (<0.1 mg g^−1)^ when using the HCDI technique. The critical contribution of nitrogen was validated through DFT analysis and post‐electrode characterizations. Furthermore, Tta‐Dfp exhibited high selectivity, achieving 79.6% for Li⁺ ions over Mg^2^⁺ ions in the electrolyte. This highlights its potential practical application in CDI devices, particularly in steps that involve the removal of competitive Mg^2^⁺ ions. Our findings emphasize the importance of molecular engineering in designing organic electrode materials for selective electrochemical resource recovery.

## 4 Experimental Section

### Electrochemical Measurement

Cyclic voltammetry (CV), galvanic charge/discharge (GCD), and electrochemical impedance spectroscopy (EIS) were performed in an AUTOLAB potentiostat (Metrohm, Switzerland). All the electrochemical experiments were carried out in 1 m LiCl aqueous electrolyte using a traditional three‐electrode system, with the Tab‐Bda, Tab‐Dfp, and Tta‐Dfp, electrode as working electrode, Ag/AgCl (saturated KCl) and Pt sheet as reference electrode and counter electrodes, respectively. The working electrodes were fabricated by first rolling the uniform mixture containing 80 wt.% active material, 10 wt.% Super P, and 10 wt.% PVDF into a sheet, and then pressing the sheet onto a Graphite sheet current collector. The specific capacitances from CV were calculated using the following equation:

(1) $$\mathrm{Cs}=\frac{\int idv}{2\times m\times \Delta V\times S}$$

where ∫ idv is the integrated area of the CV curve, m is the weight of active material (mg), ΔV is the potential window (V), and S is the scan rate (mV s^−1^).

### The CDI Performance of Tta‐Dfp COF Electrode

The LIE experiment was performed in a self‐made system. It involves a CDI unit, a peristaltic pump, a constant voltage source, and a conductivity monitor. The CDI unit consisted of a Tta‐Dfp cathode, activated carbon anode, spacers, current collectors, and end plates as a two‐electrode configuration. The dimension of the electrode was 4 × 4 cm^2,^ and the load mass of Tta‐Dfp was ≈14.5 mg. Regarding the operation, the 53 mL LiCl solution served as the target solution. It was circularly transported through the CDI unit by the constant peristaltic pump with a 20 mL min^−1^ speed. A direct voltage provided by the electrochemical workstation was applied to the CDI unit to provide electrostatic force. During the charging/discharging process, the instantaneous conductivity and current variation were detected automatically and recorded accordingly. Regarding the CDI unit, the Tta‐Dfp and AC acted as the negative and positive electrodes, respectively. The adsorption experiments of Li^+^ ions were carried out under different applied voltages (0.8–1.4 V) and different concentrations of Li^+^ ions (50, 100, 200, 300, 400, and 500 mg L^−1^ LiCl solution). Meanwhile, Li^+^ selective adsorption experiments were conducted in the mixture solution of Li^+^ and Mg^2+^ ions with different molar ratios (1:1, 1:5, and 1:10). A reverse electric field was used to carry out the desorption process of Li^+^ ions. The cation concentration in the solution was determined by an inductively coupled plasma mass spectrometry (ICP‐MS).

## Conflict of Interest

The authors declare no conflict of interest.

## Supporting information



Supporting Information

Supporting cif

## Data Availability

The data that support the findings of this study are available in the supplementary material of this article.
