# Supplementary material for: Inherited Nitrogen Distribution Control in Covalent Organic Framework Cathodes for Efficient Electrochemical Lithium Recovery via Capacitive Deionization
Source: Adv Sci (Weinh). 2025 Apr 24;12(27):2417140. doi: 10.1002/advs.202417140 (PMC12279164; doi:10.1002/advs.202417140)
Supplement: Supplementary file 1 — Supporting Information [file ADVS-12-2417140-s002.docx]

**Supplementary Information**

**Inherited Nitrogen Distribution Control in Covalent Organic Framework Cathodes for Efficient Electrochemical Lithium Recovery via Capacitive Deionization**

*Rajesh Dhanushkotti, Abdul Khayum Mohammed, Kayaramkodath Chandran Ranjeesh, Hema Mylnahalli Krishnegowda, Najat Maher Aldaqqa, and Dinesh Shetty**

Dr. R. Dhanushkotti, Dr. A. K. Mohammed, Dr. K. C. Ranjeesh, N. M. Aldaqqa, Prof. D. Shetty, Department of Chemistry, Khalifa University, PO Box 127788, Abu Dhabi, UAE.

E-mail: [dinesh.shetty@ku.ac.ae](mailto:dinesh.shetty@ku.ac.ae).

Dr. H. M. Krishnegowda, Department of Studies in Physics, Manasagangotri, University of Mysore, Mysuru, Karnataka 570 006, India

Prof. D. Shetty, Center for Catalysis & Separations (CeCaS), Khalifa University, P.O. Box 127788, Abu Dhabi, UAE.

1. Materials

4,4',4''-(1,3,5-triazine-2,4,6-triyl)trianiline (Tta) (TCI), 2,6-diformylpyridine (Dfp) (TCI), 1,3,5-tris(4-aminophenyl)benzene (Tab) (TCI), Benzene-1,4-dicarboxaldehyde (Bda) (TCI), 1, 4-dioxane (Sigma-Aldrich), 1,3,5-triformylbenzene (Tfb) (TCI), p-phenylenediamine (Pda) (TCI). mesitylene (Sigma-Aldrich), acetic acid (Merck), N, N-dimethylacetamide (DMA) (Sigma-Aldrich), acetone (Merck), were used as received. All the reactions were carried out in oven-dried 100 ml heavy walled (HW) pressure glass vessel capped with a Teflon screw cap with rubber internal thread under an air atmosphere unless otherwise mentioned.

2. Synthesis

2.1 Synthesis of Tta-Dfp:

We synthesized Tta-Dfp by using the Schiff base condensation reaction with 2, 6-diformylpyridine (Dfp) (11.43 mg, 0.084 mmol) and 4, 4′, 4′′-(1, 3, 5-triazine-2,4,6-triyl) trianiline (Tta) (20 mg, 0.056 mmol) in 2 mL of a 1, 4-dioxane/mesitylene mixture (1:1 v/v). A catalytic volume of acetic acid (50 µl, 6 M) was used in a sealed pressure tube to perform the reaction. After five days of heating at 120 °C, we collected the yellow precipitate by centrifuging it. We repeatedly cleaned it with acetone, water, and N, N'-dimethylacetamide (DMA) to eliminate residues and impurities. The sample dried at 80 °C for 12 hours under vacuum. We obtained an isolated yield of 82% (26 mg) of Tta-Dfp COF as a yellow solid.


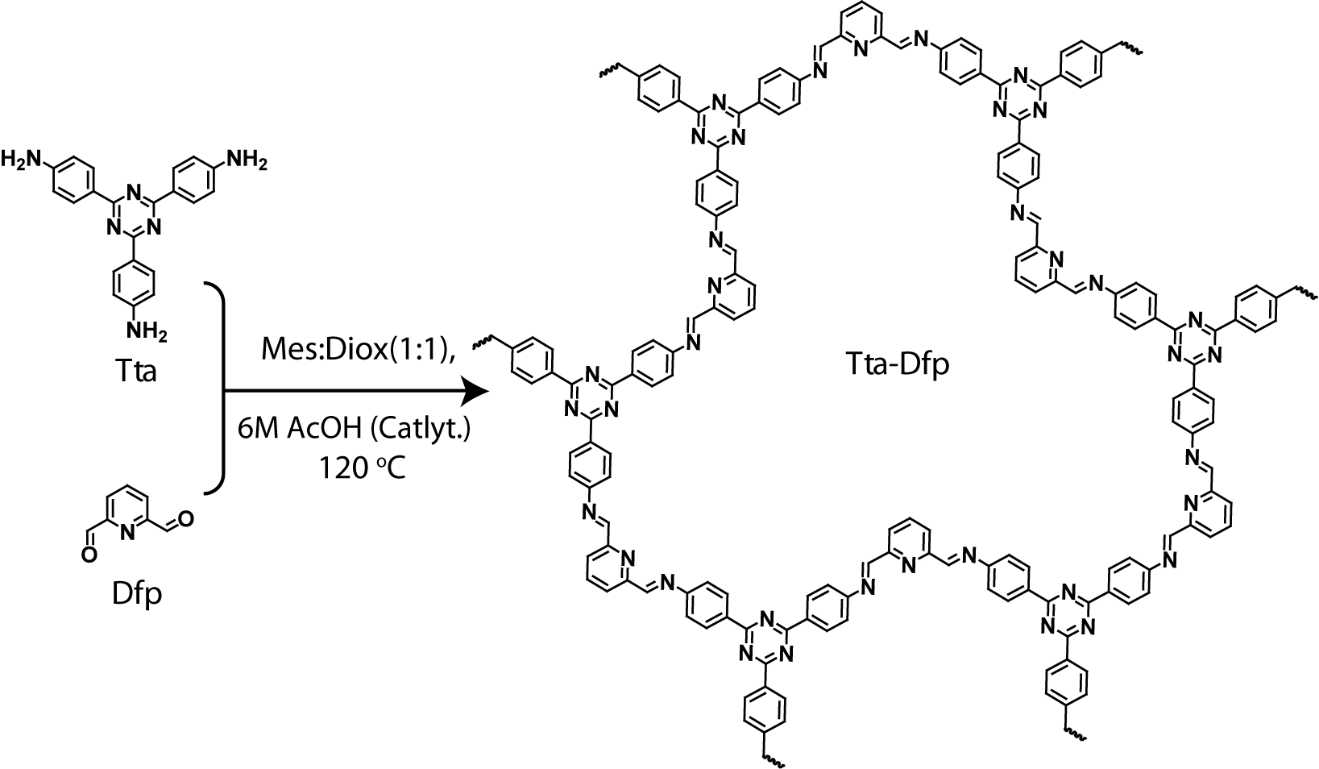


Scheme S1: The Tta-Dfp synthetic scheme.

2.2 Synthesis of Tab-Dfp:

Tab-Dfp was synthesized using a Schiff base condensation reaction by mixing 2,6-diformylpyridine (Dfp) (11.53 mg, 0.085 mmol) with 1,3,5-tris(4-aminophenyl) benzene (Tab) (20 mg, 0.056 mmol). The reaction occurred in a solution of 2 mL of 1,4-dioxane and mesitylene, with a 1:1 volume ratio. The experiment was conducted in a pressure vessel that was completely sealed, using a small amount of acetic acid (50 µl, 6M) as a catalyst. After undergoing a heating process at a temperature of 120 °C for a period of five days, the resulting yellow solid was separated using centrifugal force. It was then subjected to several washes with N, N-dimethylacetamide (DMA), water, and acetone to remove impurities and unreacted substances. Afterward, the sample was dried at 80 °C for 12 hours in a vacuum. That process yielded the formation of Tab-Dfp COF, which materialized as a yellow solid with a 79% isolated yield (25 mg).


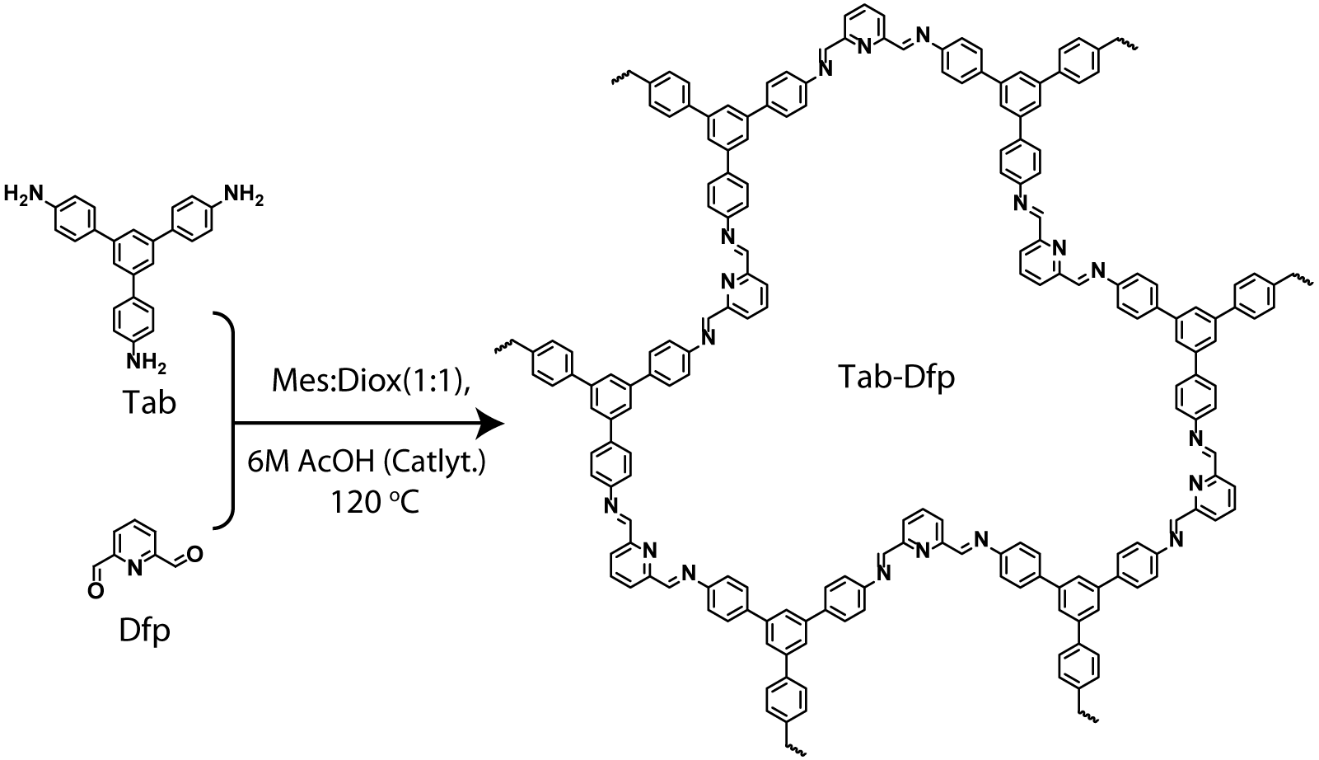


Scheme S2: The Tab-Dfp Synthetic scheme.

2.3 Synthesis of Tab-Bda:

Tab-Bda was produced by reacting 1,3,5-tris(4- aminophenyl) benzene (Tab) 20 mg, 0.056 mmol) with Benzene-1,4-dicarboxaldehyde (Bda) (11.45 mg, 0.085 mmol) in 2 mL of 1, 4-dioxane/mesitylene mixture (1:1v/v) via Schiff base condensation. The reaction was carried out in a sealed pressure tube using 50 µl, 6M catalytic acetic acid. After five days at 120 °C, the yellow 4 precipitate was collected by centrifuging. It was then repeatedly cleaned using N, N-dimethylacetamide (DMA), water, and acetone to remove impurities and unreacted products. After the sample was vacuum-dried for 12 hours at 80 °C, a yellow material comprising 76% (24 mg) of the isolated yield of Tab-Bda COF was recovered.


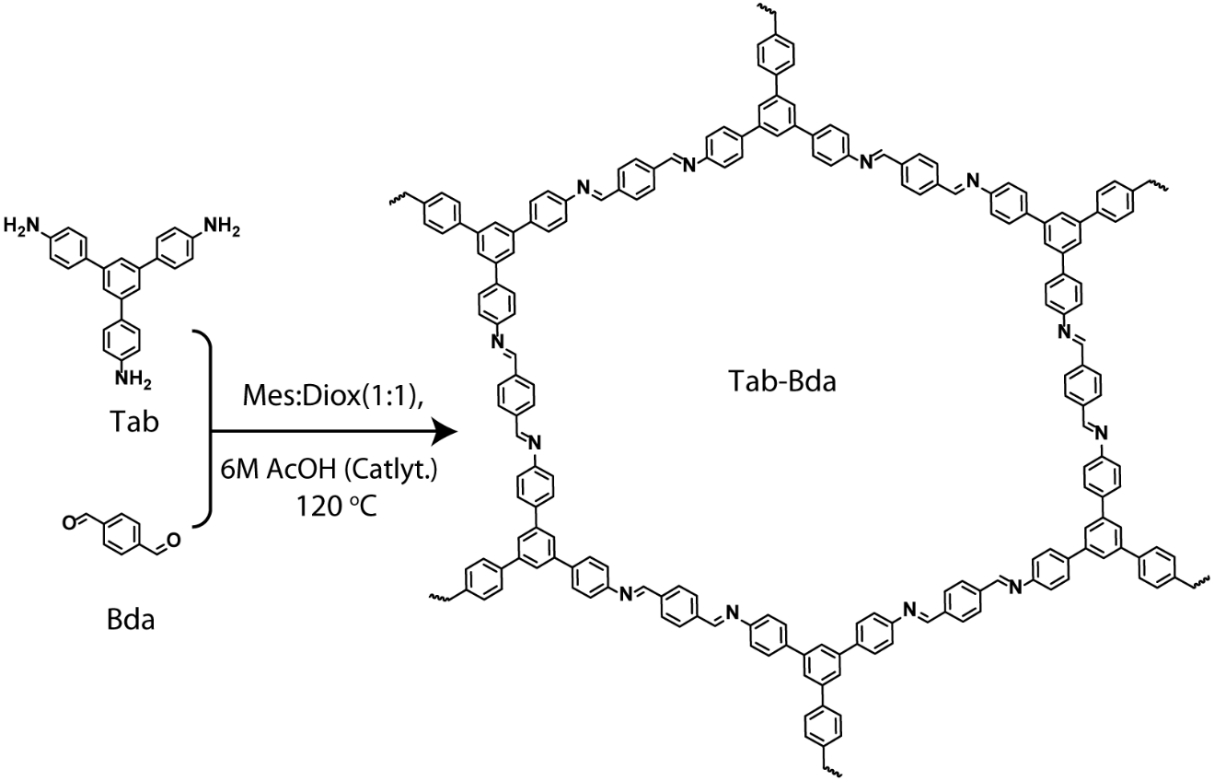


Scheme S3: The Tab-Bda synthetic scheme.

2.4 Synthesis of Tfb-Pda COF

Tfb-Pda COF was synthesized via Schiff base condensation between 1,3,5-triformylbenzene (Tfb) and p-phenylenediamine (Pda). Briefly, Tfb (20 mg, 0.123 mmol) and Pda (20.01 mg, 0.185 mmol) were dissolved in 2 mL of a 1:1 (v/v) mixture of 1,4-dioxane and mesitylene in a sealed pressure tube. 50 µL of 6 M acetic acid was added to catalyze the reaction, and the mixture was heated at 120 °C for 5 days. Upon completion, a dark yellow precipitate was collected by centrifugation, followed by extensive washing with N,N-dimethylacetamide (DMA), water, and acetone to remove residual impurities and unreacted monomers. The purified solid was then vacuum-dried at 80 °C for 12 hours, yielding Tfb-Pda COF as a dark yellow powder (72% yield, 29 mg).

Scheme S4: The Tfp-Pda synthetic scheme.

3. Instruments and methods

Powder X-ray diffraction (PXRD): Powder X-ray diffraction measurements were performed on Rigaku Smart Lab II with Cu Kα (λ = 1.5405 Å) radiation source operating at 40 kV and 40 mA. The patterns were recorded with a divergent slit of 1/16° over the 2Ɵ range of 2–50° with step size = 0.02°.

Fourier transform infrared spectroscopy (FT-IR): FT-IR spectra were taken on a Bruker Optics ALPHA-E spectrometer with a universal Zn-Se ATR (attenuated total reflection) accessory in the 600- 4000 cm^-1^ region or using a Diamond ATR (Golden Gate) with 24 scan rate and 4 cm^-1^ resolution.

The morphology of the materials was characterized by scanning electron microscopy (SEM, JEOL JSM-7610F FEG-SEM. The SEM samples were prepared by drop-casting 10 μL of COFs slurry (COFs dispersed in Isopropyl alcohol) on a silicon substrate and dried in air followed by Pt coating (nano-sized film) using the JEOL JEC-300FC Auto Fine before SEM analysis. Further, transmission electron microscopy (TEM) and high-resolution TEM (HR-TEM) were employed for the in-depth morphological analysis by FEI Tecnai TEM 20 kV. The TEM samples were prepared by drop casting the COFs 7 dispersion (dispersed in Isopropyl alcohol) over carbon grids (TED PELLA, INC. 200 mesh) and allowed to dry overnight in desiccators.

X-ray photoelectron spectroscopy (XPS): XPS measurements were performed using a Supra^+^ instrument (Kratos, Manchester, UK) equipped with an Al Kα excitation source and a monochromator. The charge neutralizer was on during the measurements. The take-off angle was 90°. XPS measurements and data processing were performed using ESCApe 1.5 software (Kratos). The powder samples were placed on a carbon tape attached to the silicon wafer. The area analysed was 300 by 700 microns. The measurements were performed at a pass energy of 20 eV. The base pressure in the main analysis chamber was 8·10–8 mbar. The binding energy scale was corrected based on the C-C/C-H peak at 284.8 eV in the C 1s spectrum.

4. Calculation of electrosorption performances

The LIE capacity was calculated

$Q=\frac{\left( C_{0}-C_{e} \right)\times V}{m}$ (Equation S1)

where *C_o_* and *C_e_* (mol L^-1^) are the ion concentrations in the solution before and after CDI adsorption, respectively; *V* (L) is the solution volume and *m* is the mass of the active materials.

The Li^+^ electrosorption rates (R, mol∙L ^-1^∙min^-1^) could be evaluated by the Kim-Yoon plot:

$R = Q/t$ (Equation S2)

where 𝑄 (mg.g ^-1^ ) represents the electrosorption capacitance; t (min) is the electrosorption time.

The selective lithium performance was calculating the separation factor (α ^Li+^_Mg_^2+^) of Li+ over Mg^2+^

$Adsorption ratio=\left( C0-Ce \right)/C0*100$ (Equation S3)

Where C0 and Ce are the initial and final concentrations of lithium ions and magnesium ions, respectively. The unit for all cation concentrations is mol L^-1^.

Lithium recovery efficiency is calculated by following equation:^[1]^

$E\left( \% \right)=\frac{\left( Recovered lithium amount \right)}{\left( Initial lithium amount in solution \right)}*100$ (Equation S4)

5. Density Functional Studies

All density-functional theory (DFT) calculations were conducted using the Gaussian 16 software package and visualized with Gauss View 6^[2]^. Specifically, Gaussian 16 was employed to optimize the geometries and frontier molecular orbitals. The B3LYP/6-31G(d) level of theory was used for carbon, nitrogen, and hydrogen atoms, while the B3LYP/LanL2DZ basis set was applied for the Li ion ^[3]^. Based on the optimized electron density wave function, molecular electrostatic potential analysis was performed to evaluate the molecule's electrostatic potential (ESP). The electron localization function (ELF) calculations were carried out using the Multiwfn 3.7 program^[4]^.


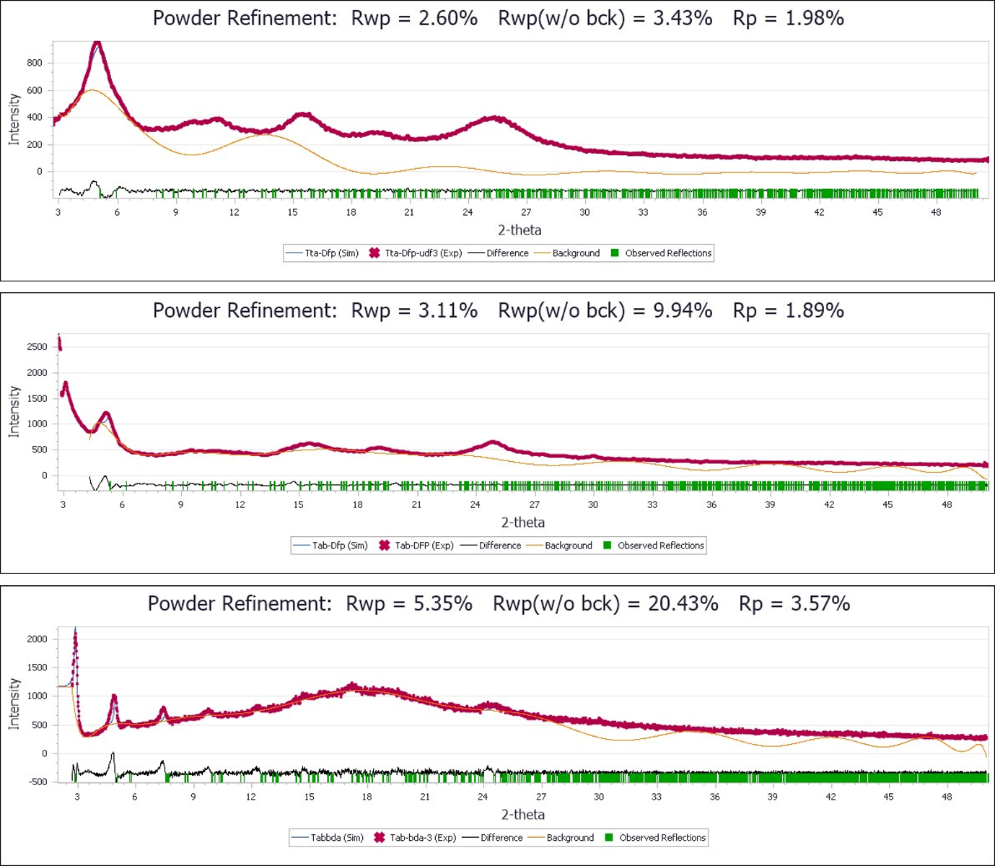


Figure S1: Comparison of experimental PXRD pattern of Tta-Dfp, Tab- Dfp, and Tab-Bda (-) with corresponding simulated, and Pawley refined difference; Background; inset shows Rp, Rwp, and Rwp (w/o bck) (lattice parameters: R, weighted profile R values: Rwp, Un-weighted profile R values Rp)


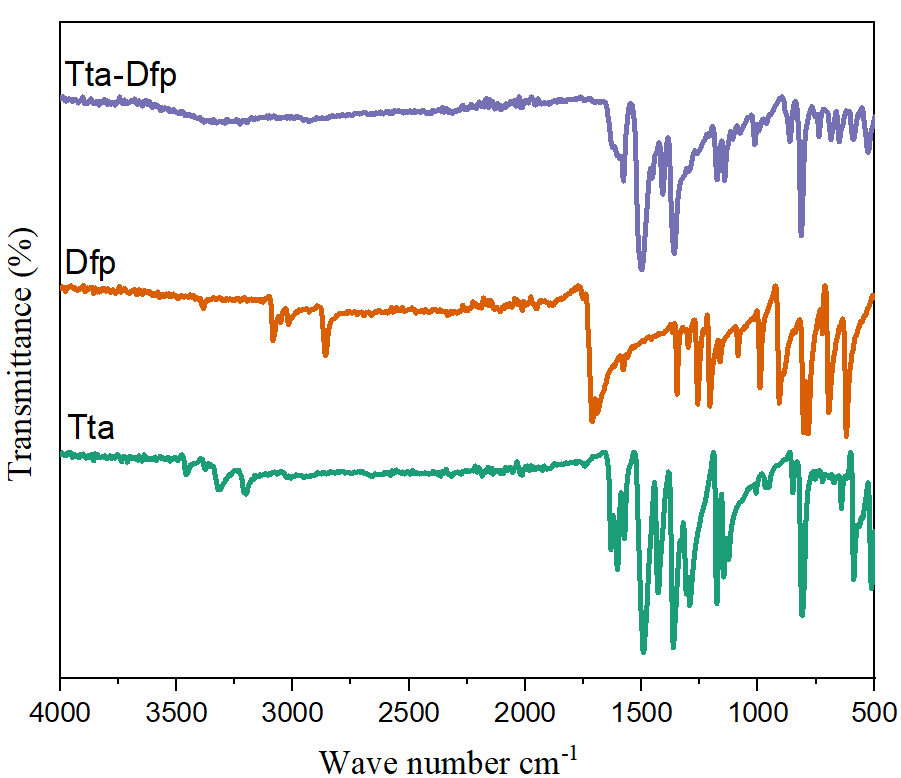


Figure S2: FT-IR spectra of Tta-Dfp


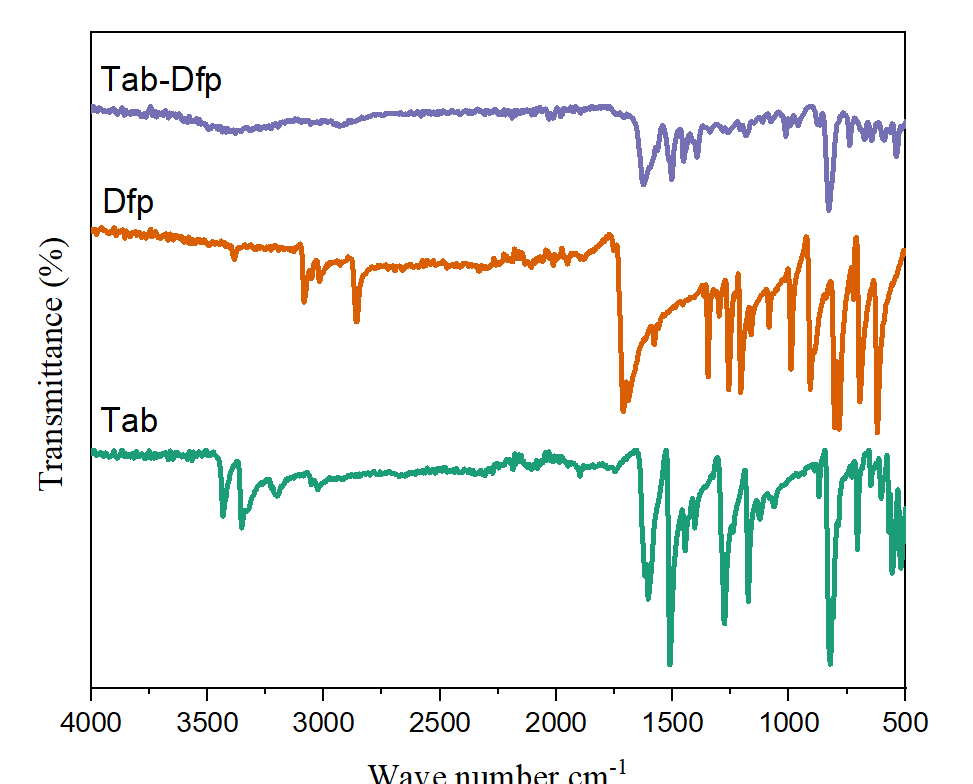


Figure S3: FT-IR spectra of Tab-Dfp


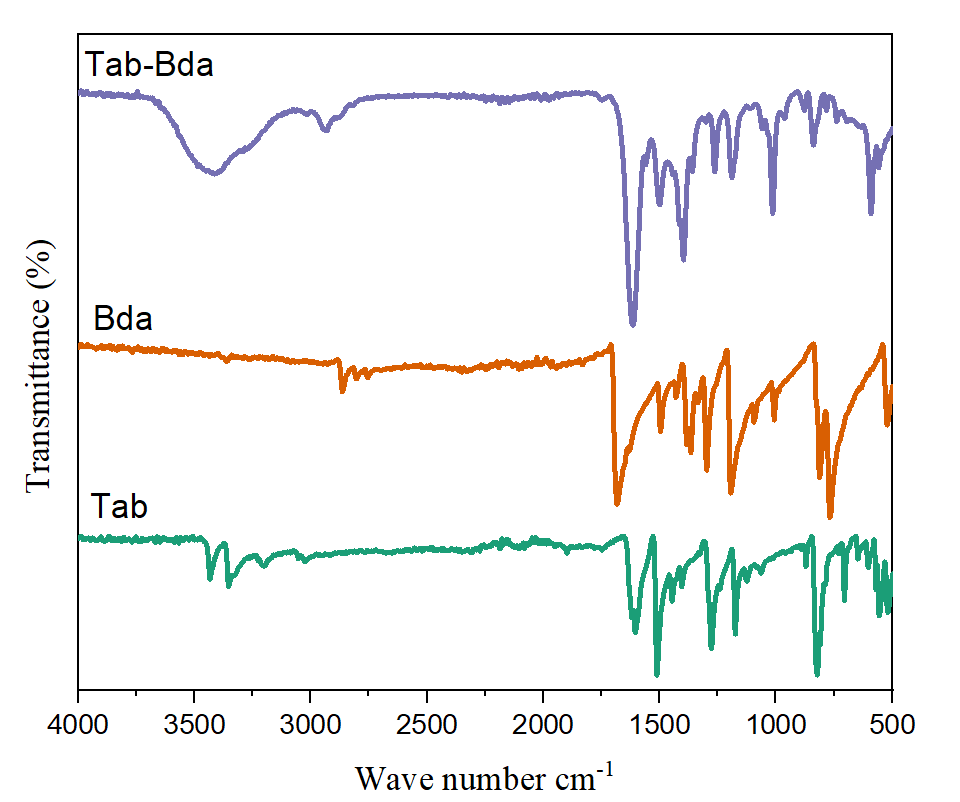


Figure S4: FT-IR spectra of Tab-Bda


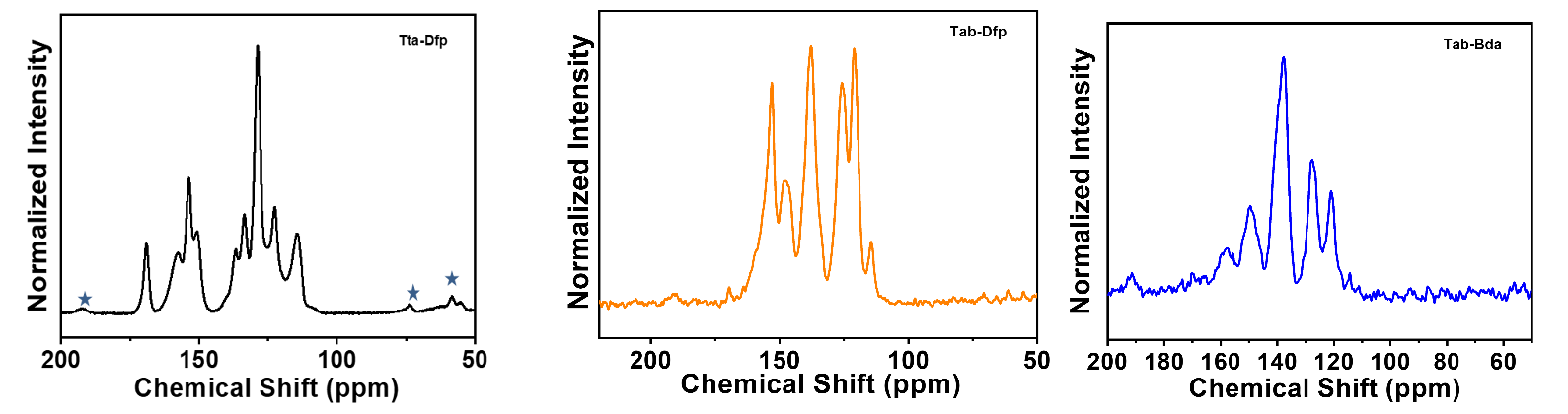


Figure S5: ^13^C NMR for Tta-Dfp, Tab-Dfp, and Tab-Bda COF; asterisks denote spinning sidebands


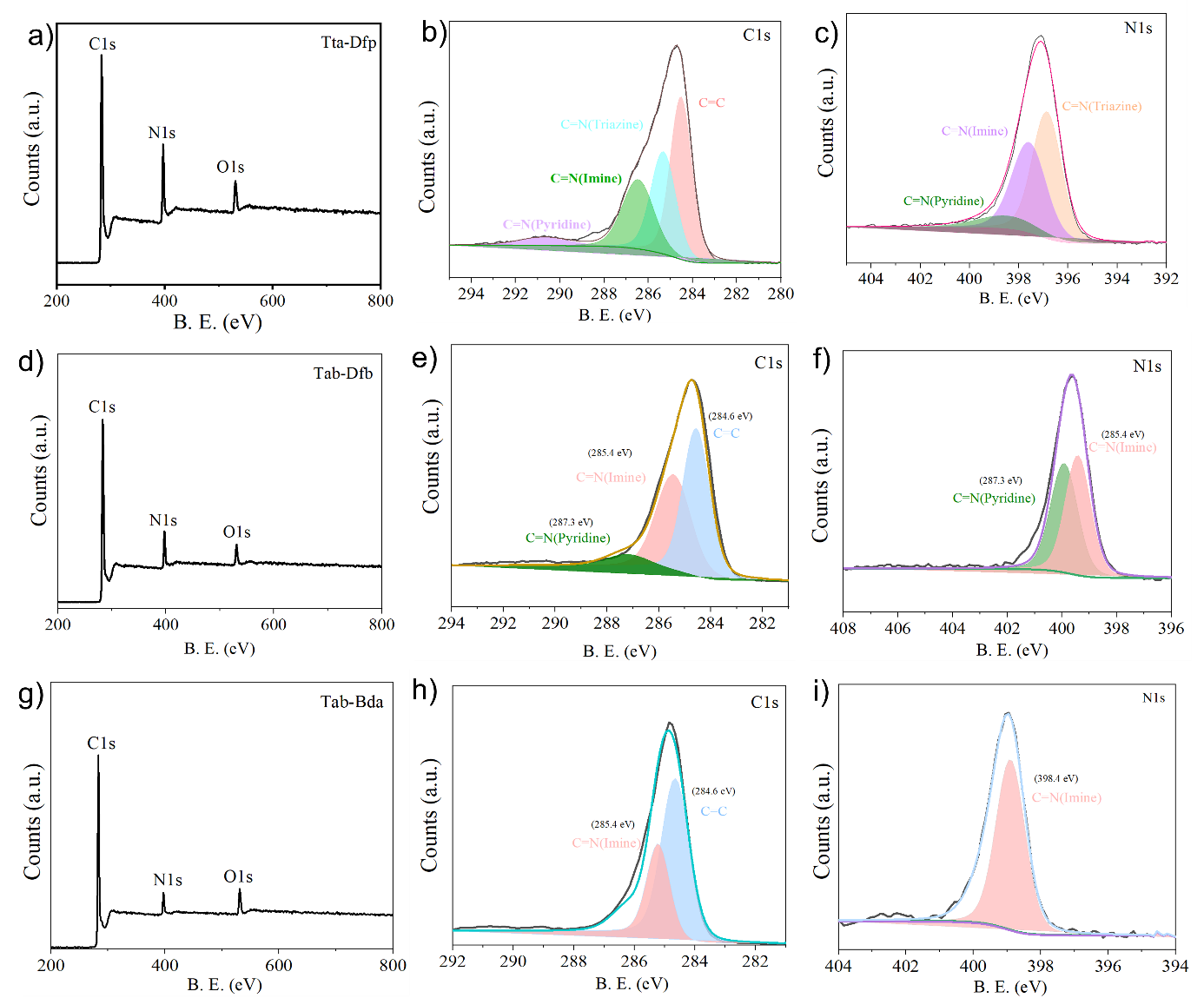


Figure S6: XPS spectrum of a) wide spectrum, b) C 1s spectra, c) N 1s spectra of X-ray photo spectroscopy of Tta-Dfp COF, d) wide spectrum, e) C 1s spectra, f) N 1s spectra of X-ray photo spectroscopy of Tab-Dfp COF, g) wide spectrum, h) C 1s spectra, i) N 1s spectra of X-ray photo spectroscopy of Tab-Bda COF


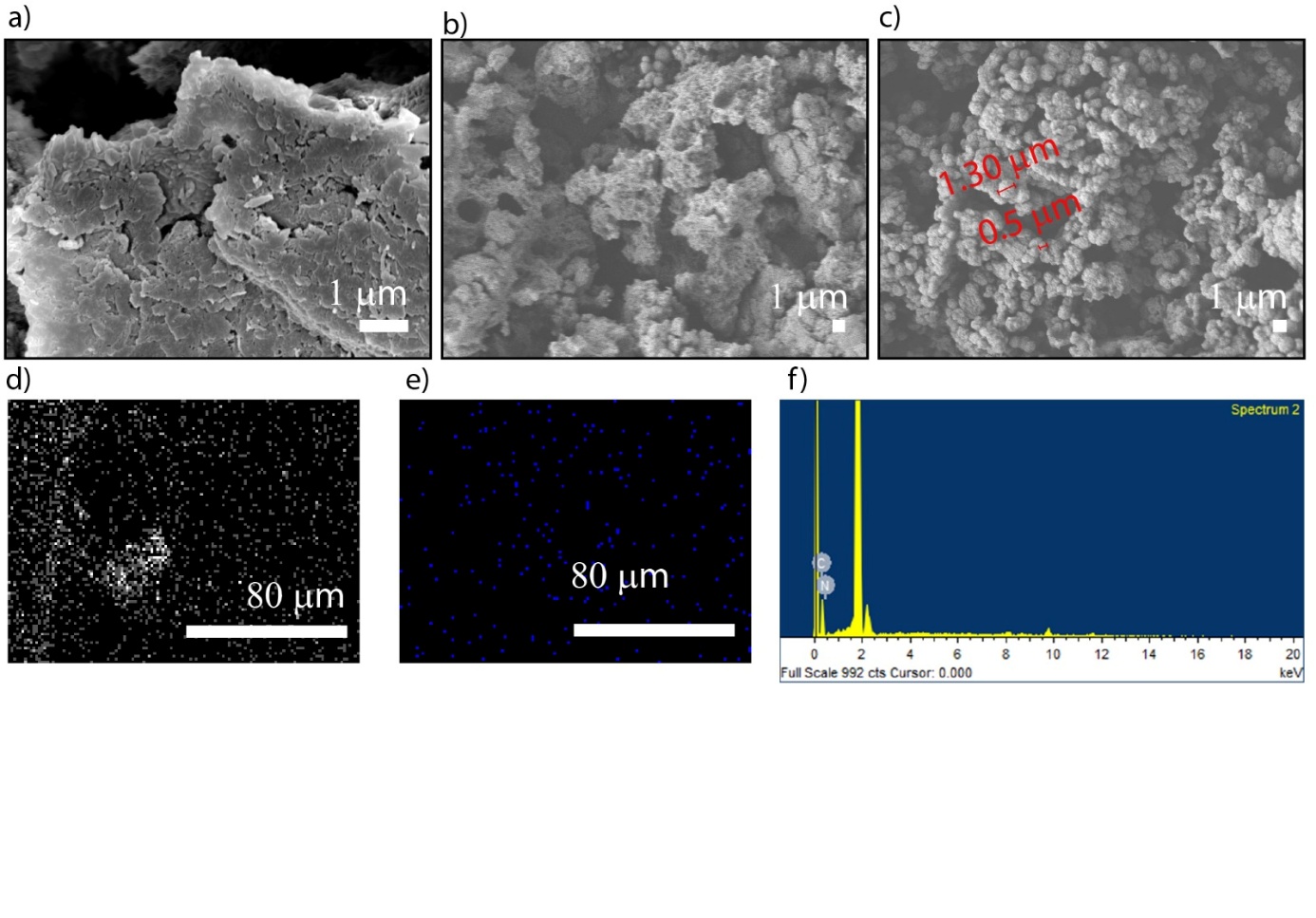


Figure S7: SEM image of a) Tta-Dfp, b) Tab-Dfp, and c) Tab-Bda, d) & e) SEM-EDS elemental dot mapping of Tta-Dfp and f) corresponding EDS spectra of Tta-Dfp


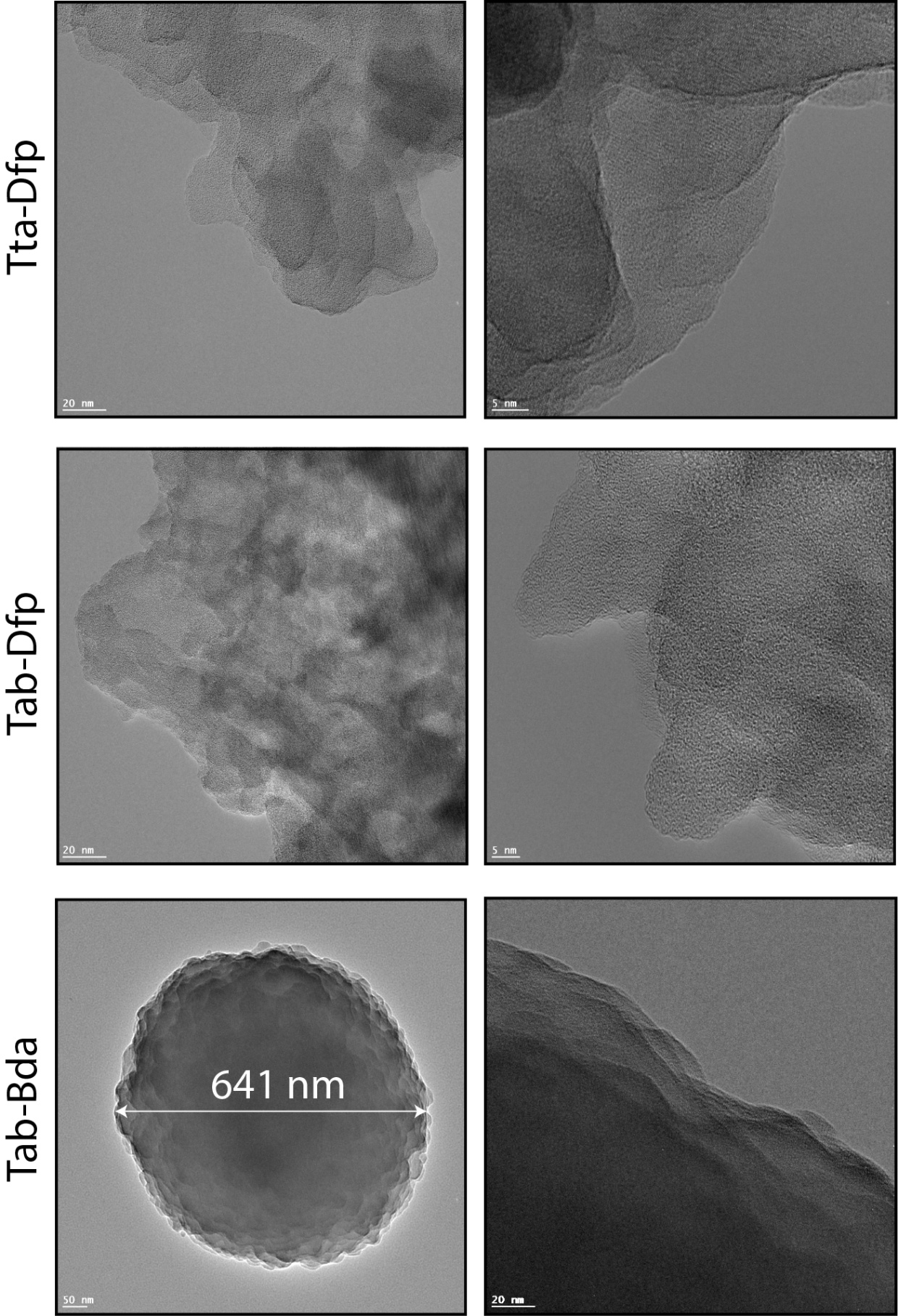


Figure S8: The TEM images of COFs

Figure S9: The N_2_ adsorption isotherms and pore size distribution profiles of a-b) Tta-Dfp; c-d) Tab-Dfp; and e-f) Tab-Bda.


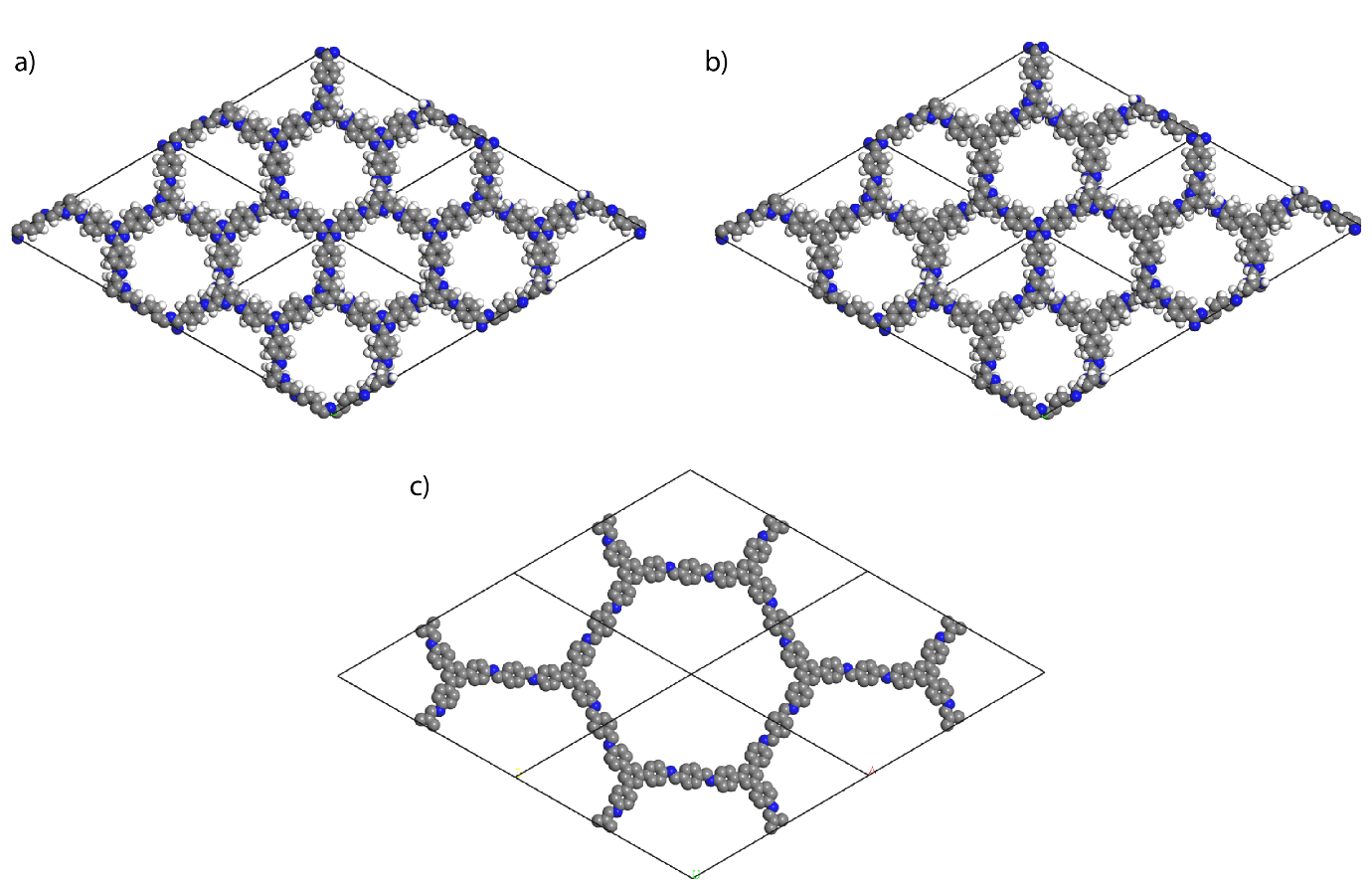


Figure S10: The theoretical models of COFs- a) ABC stacking in Tta-Dfp; b) ABC stacking in Tab-Dfp; and c) AA stacking in Tab-Bda.


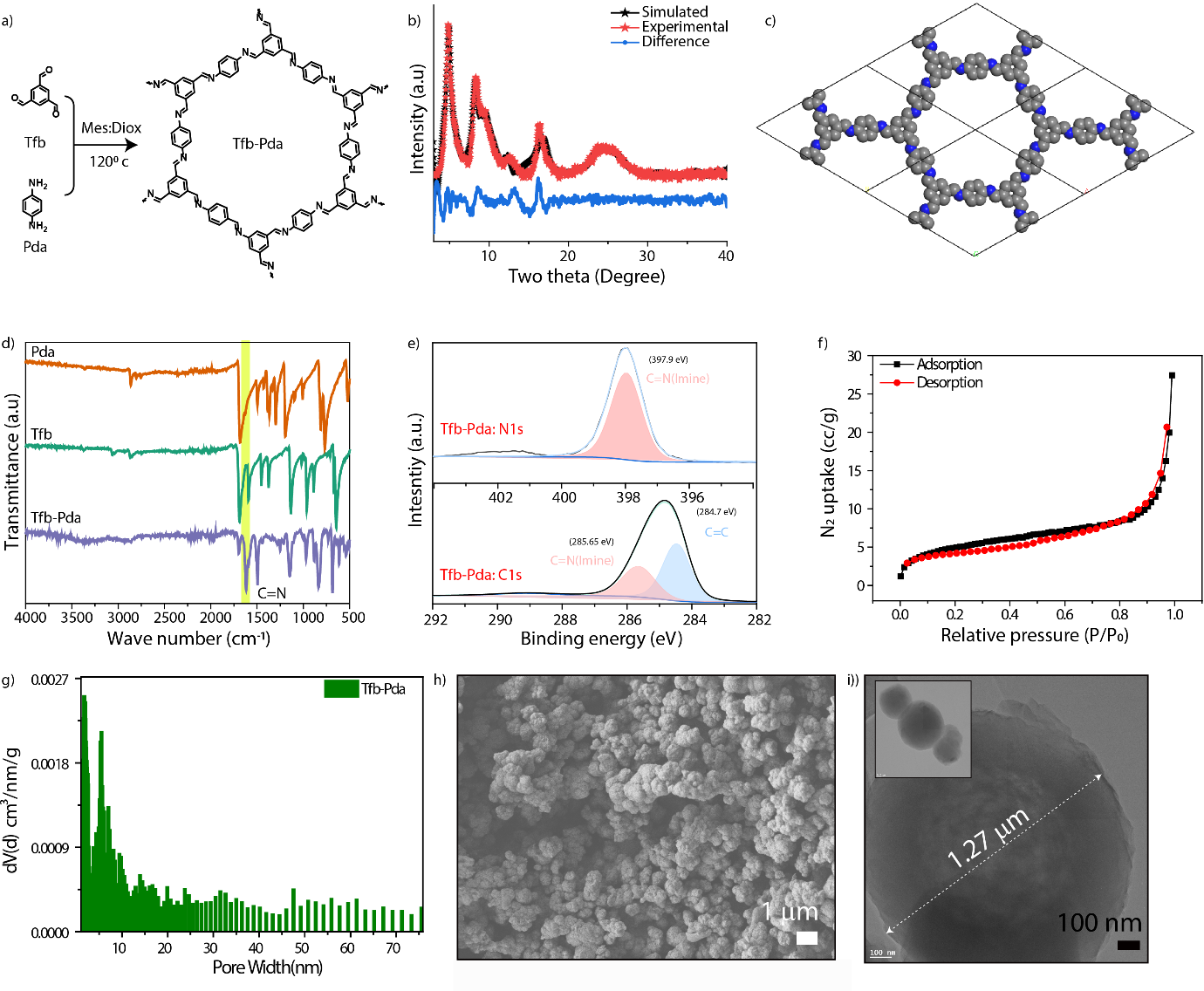


Figure S11: The synthesis and characterizations of Tfb-Pda. a) The schematic representation of the synthesis Tfb-Pda; b) the PXRD profiles; c) the theoretical AA-stacking model; d) the FT-IR; e) XPS profiles C1s and N1s, f) the N_2_ gas adsorption isotherm; g) the pore size distribution; h) the SEM image and i) the TEM image (inset: the TEM image of the scale bar 200 nm) of Tfb-Pda

Figure S12: a-b) Cyclic voltammetric curves of the Tta-Dfp and Tab-Dfp electrodes at various scan rates of 1–50 mV s^−1^, c-d) GCD curves of the Tta-Dfp and Tab-Dfp electrodes at various current densities of 0.1 to 1 A g^−1^


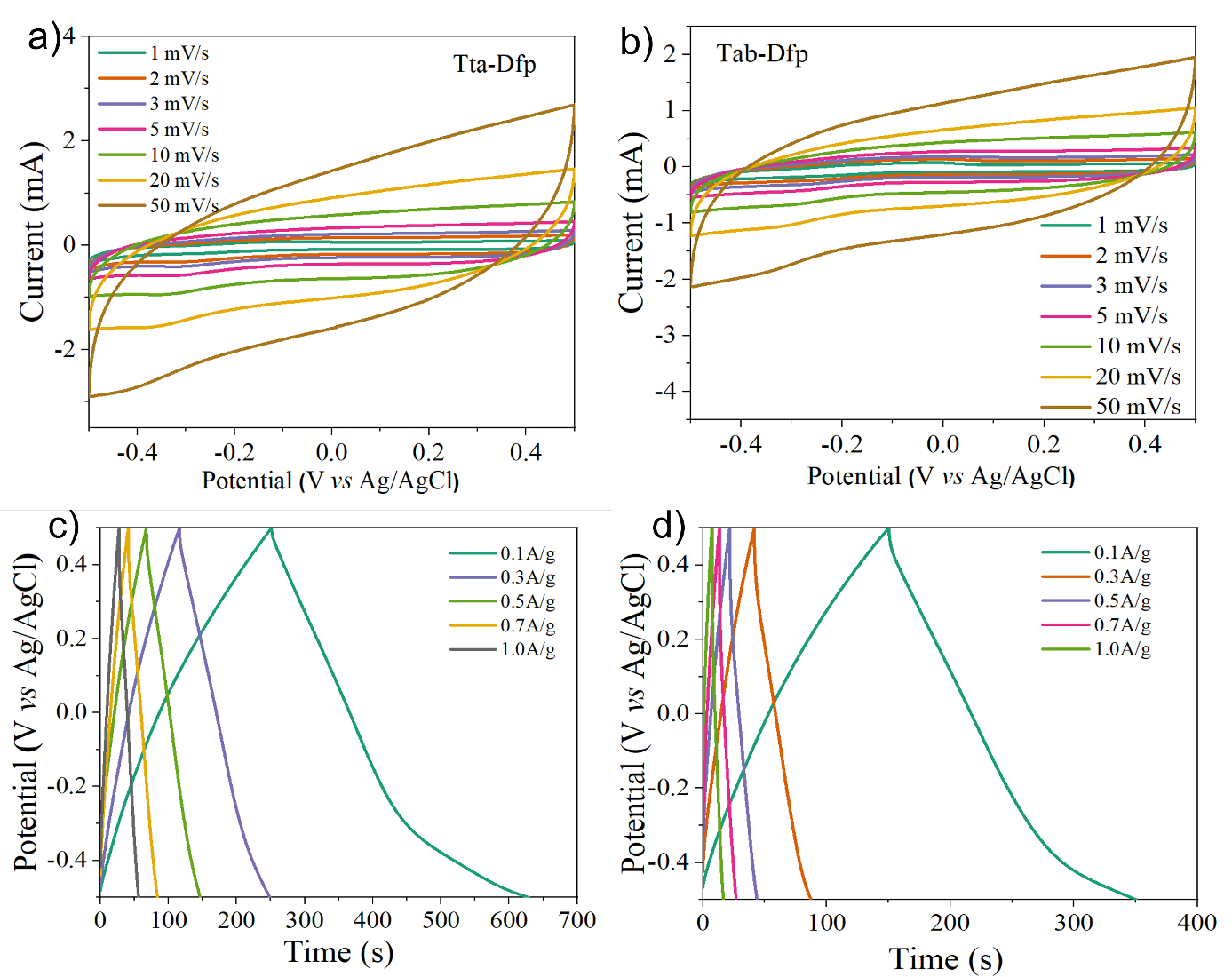


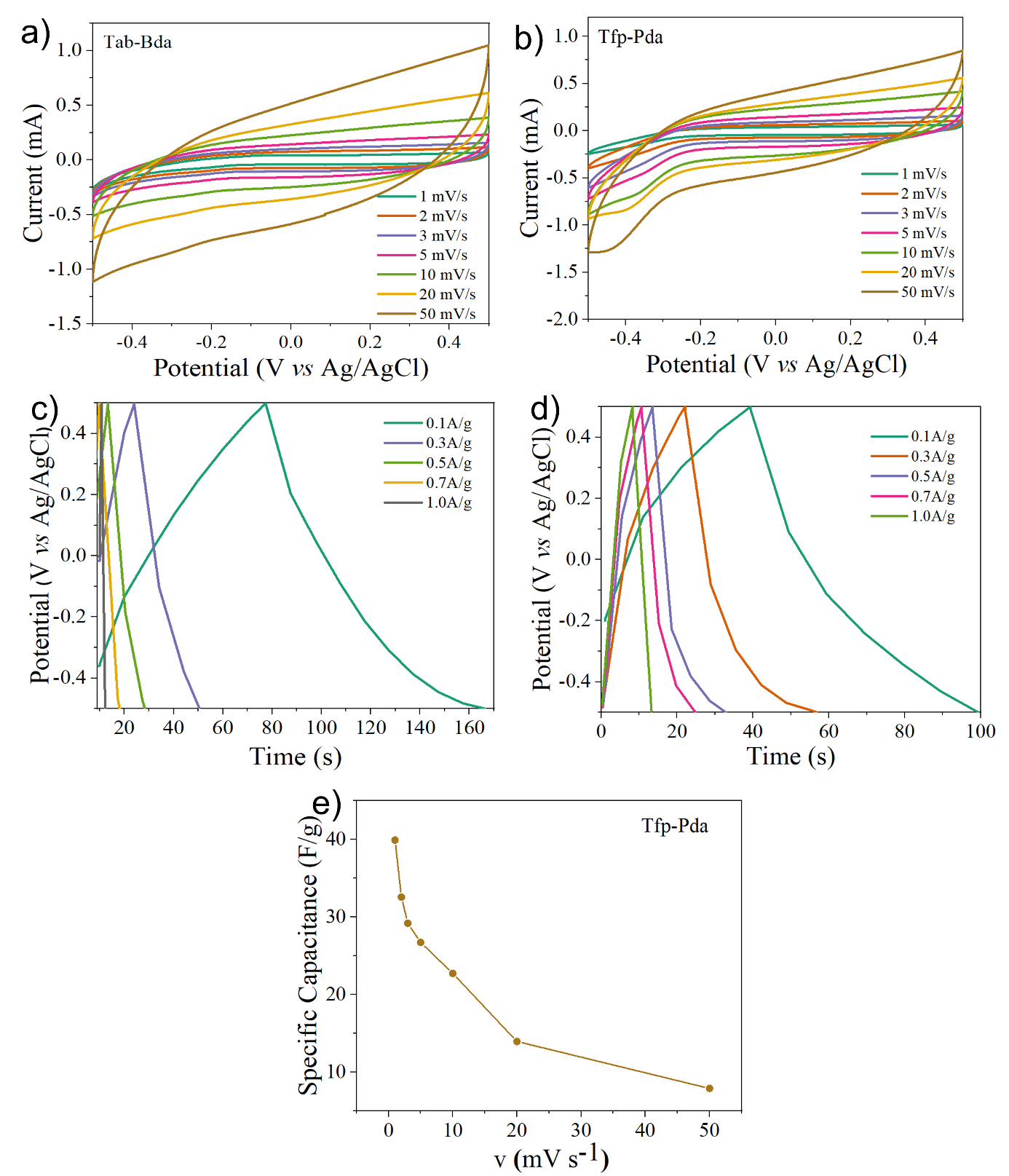
Figure S13: a-b) Cyclic voltammetric curves of the Tab-Pda and Tfb-Pda electrodes at various scan rates of 1–50 mV s^−1^, c-d) GCD curves of the Tab-Pda and Tfb-Pda electrodes at various current densities of 0.1 to 1 A g^−1^


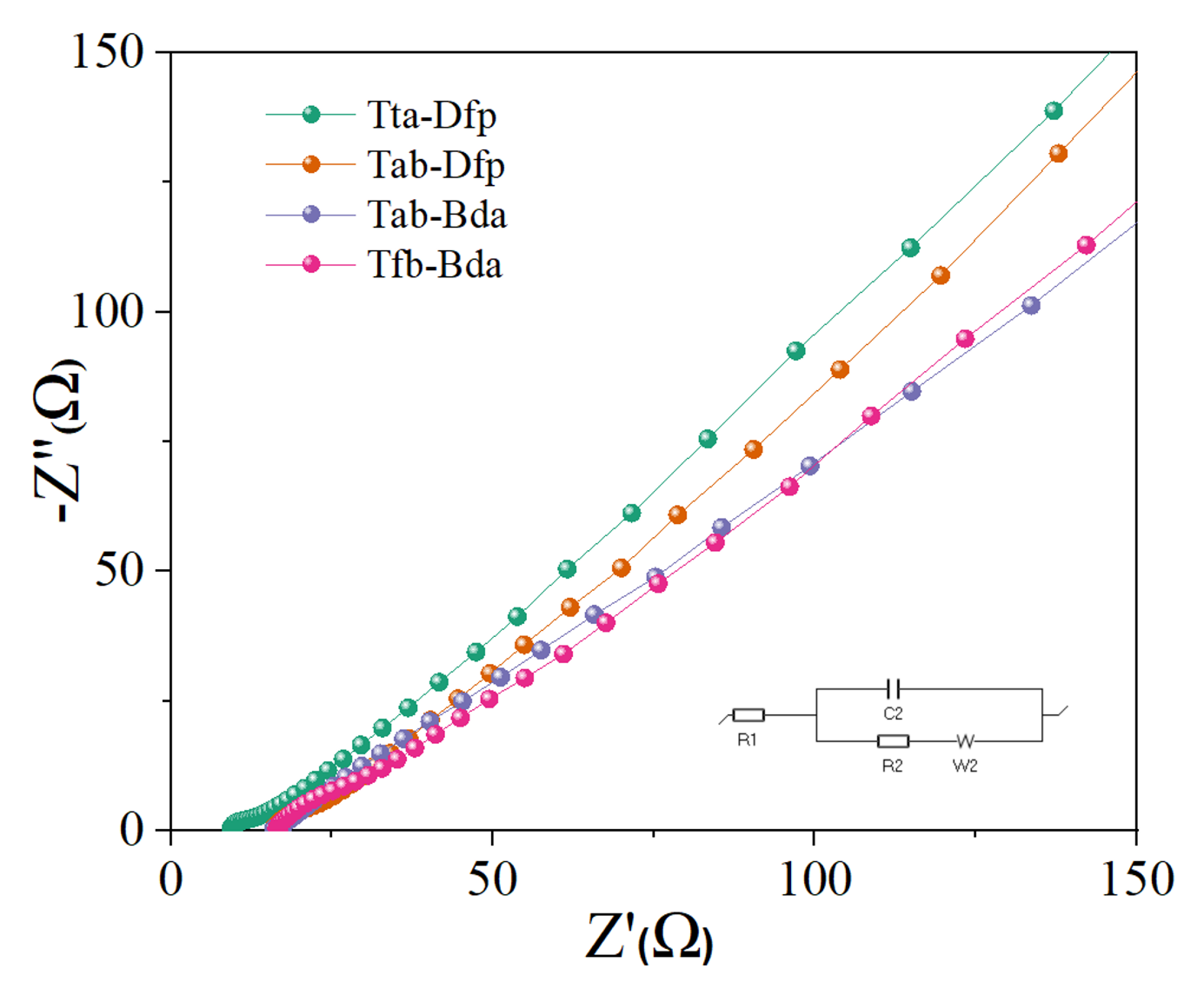


Figure S14: The EIS plots of all COFs.


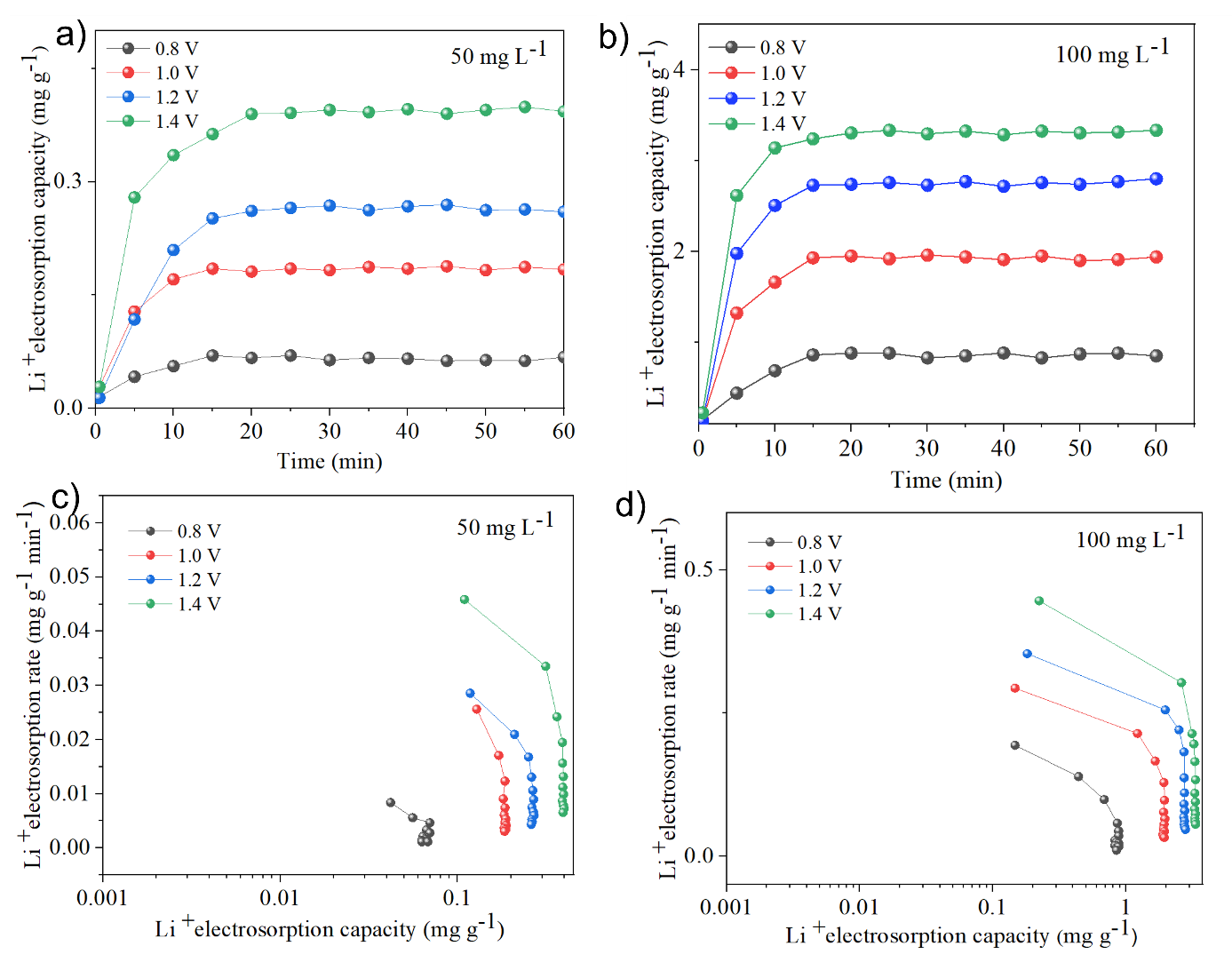


Figure 15: a-b) Electrosorption capacity, c-d) Kim–Yoon plot of the Tta-Dfp electrode under the different potential of Li^+^ ions solution at different potential in 50 mg L^−1^ and 100 mg L^−1^


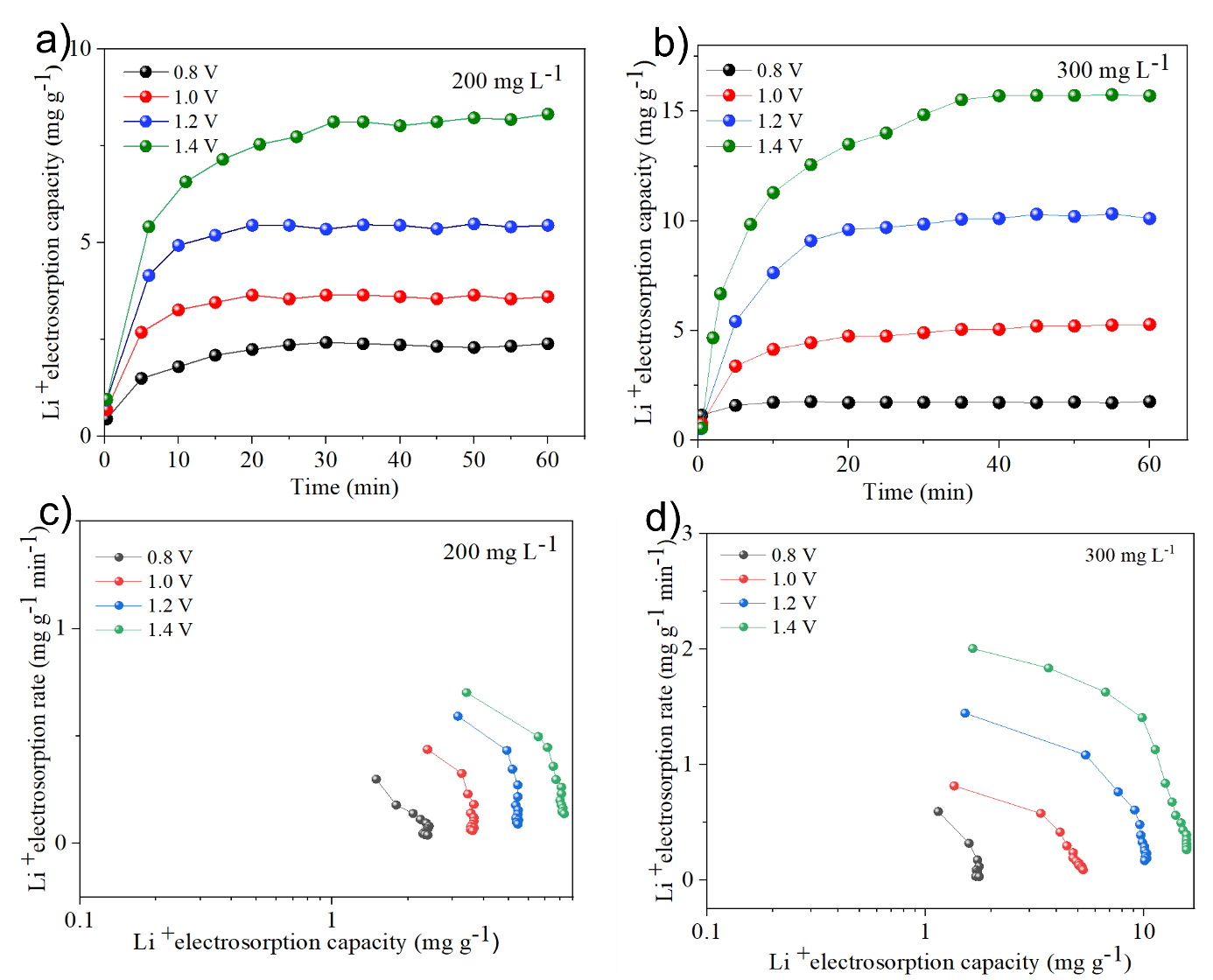


Figure 16: a-b) Electrosorption capacity, c-d) Kim–Yoon plot of the Tta-Dfp electrode under the different potential of Li^+^ ions solution at different potential in 200 mg L^−1^ and 300 mg L^−1^


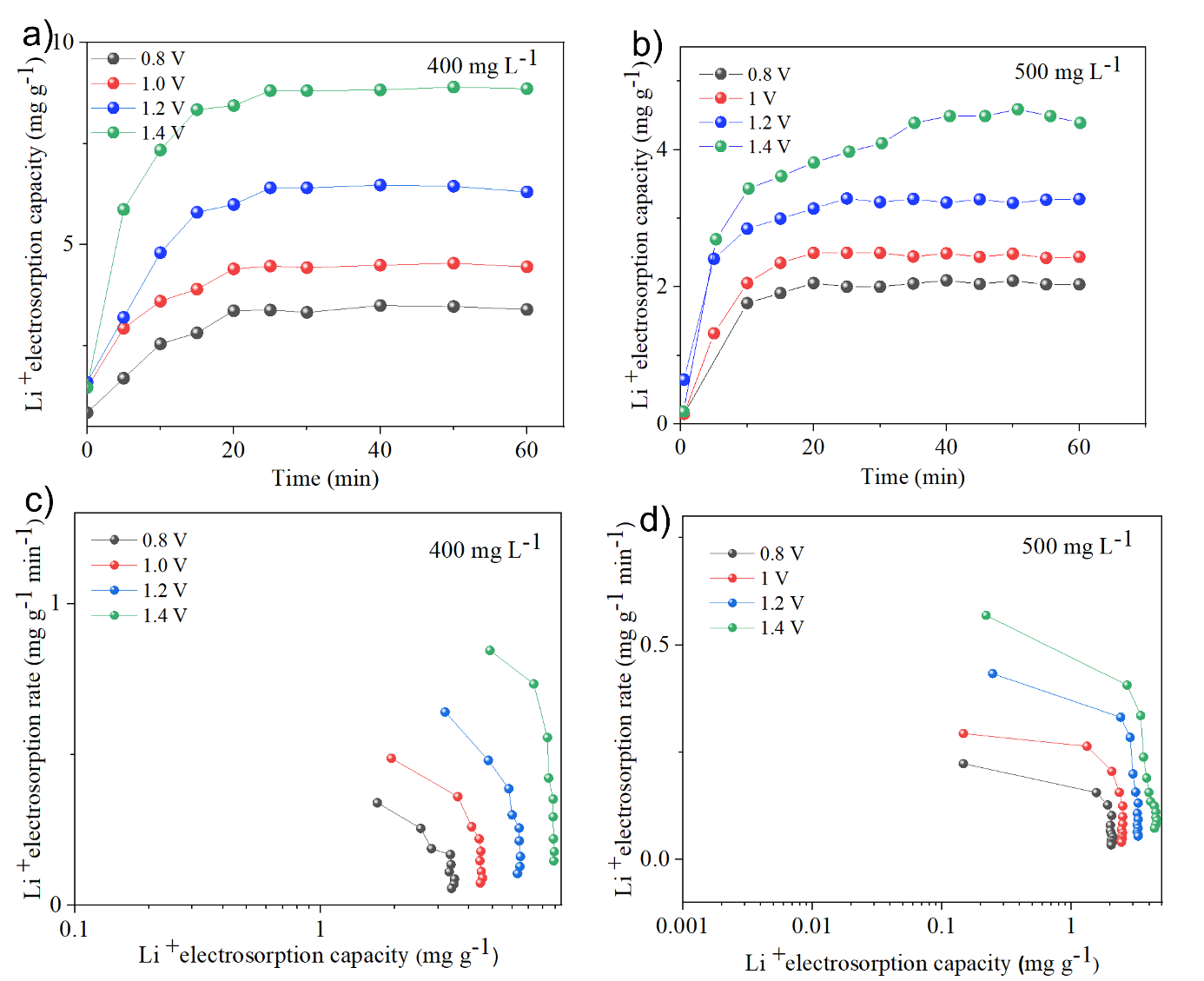


Figure 17: a-b) Electrosorption capacity, c-d) Kim–Yoon plot of the Tta-Dfp electrode under the different potential of Li^+^ ions solution at different potential in 400 mg L^−1^ and 500 mg L^−1^


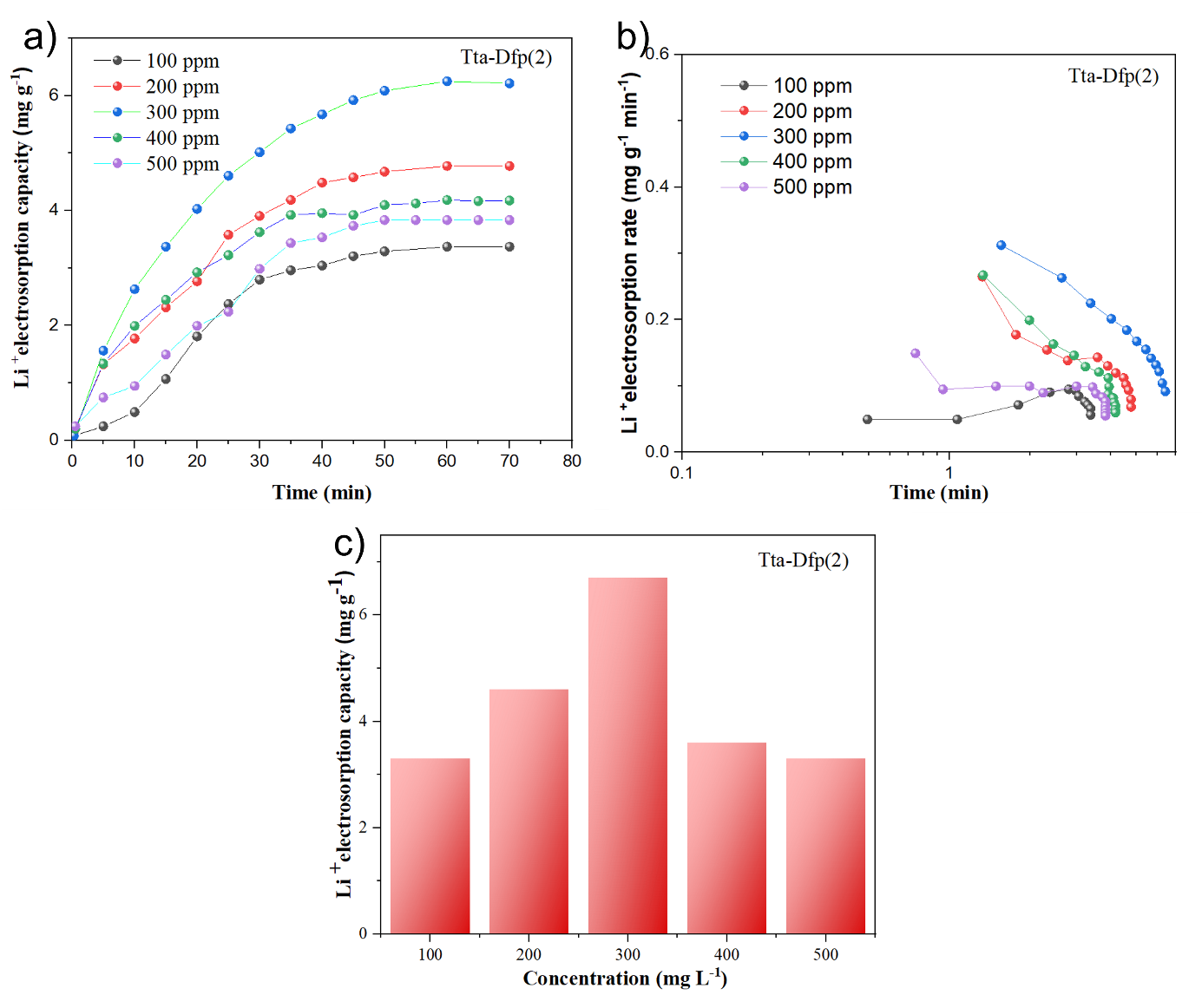


Figure S18: Tta-Dfp-2 (26 mg) electrode tested with difference concentration at difference concentration


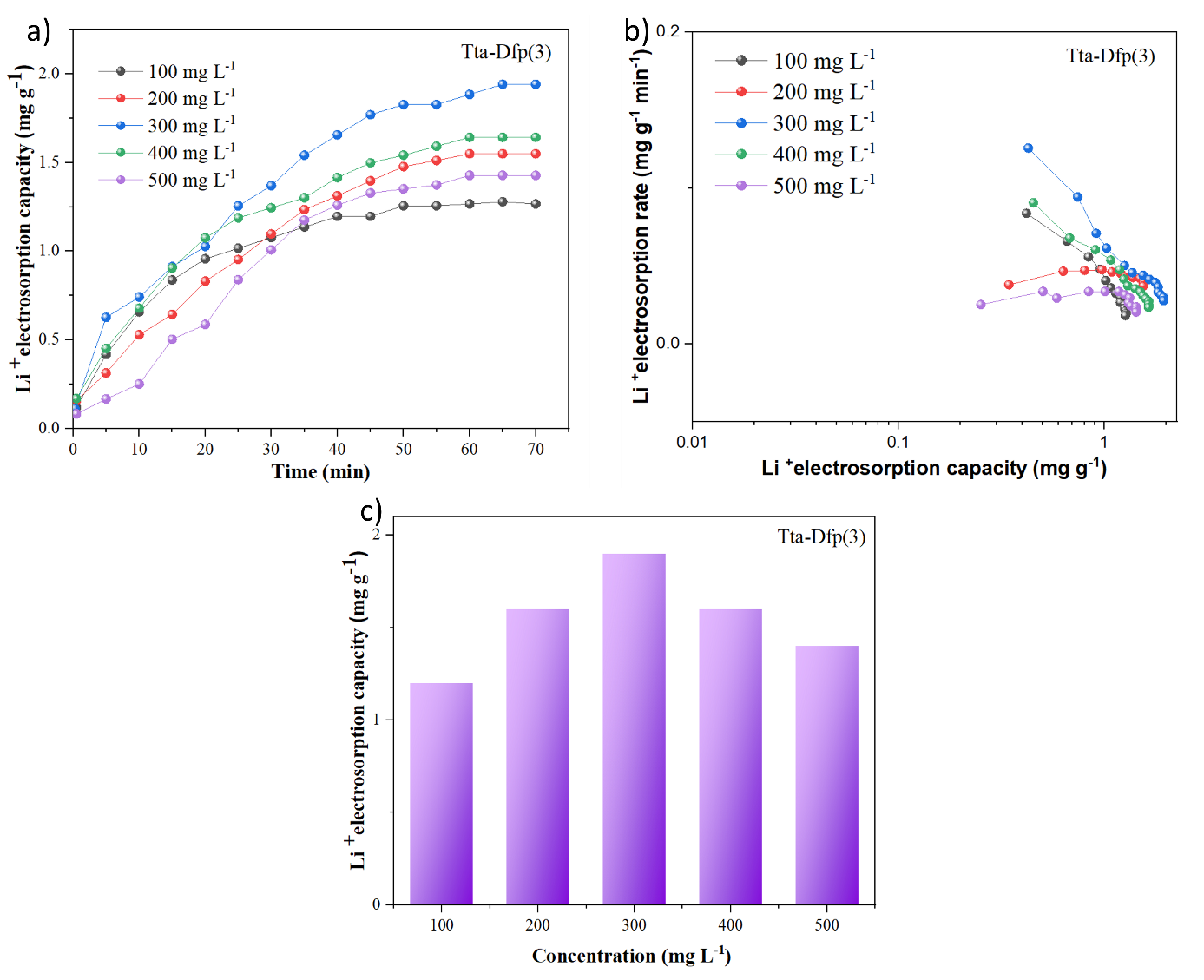


Figure S19: Tta-Dfp-3 (35 mg) electrode tested with difference concentration at difference concentration


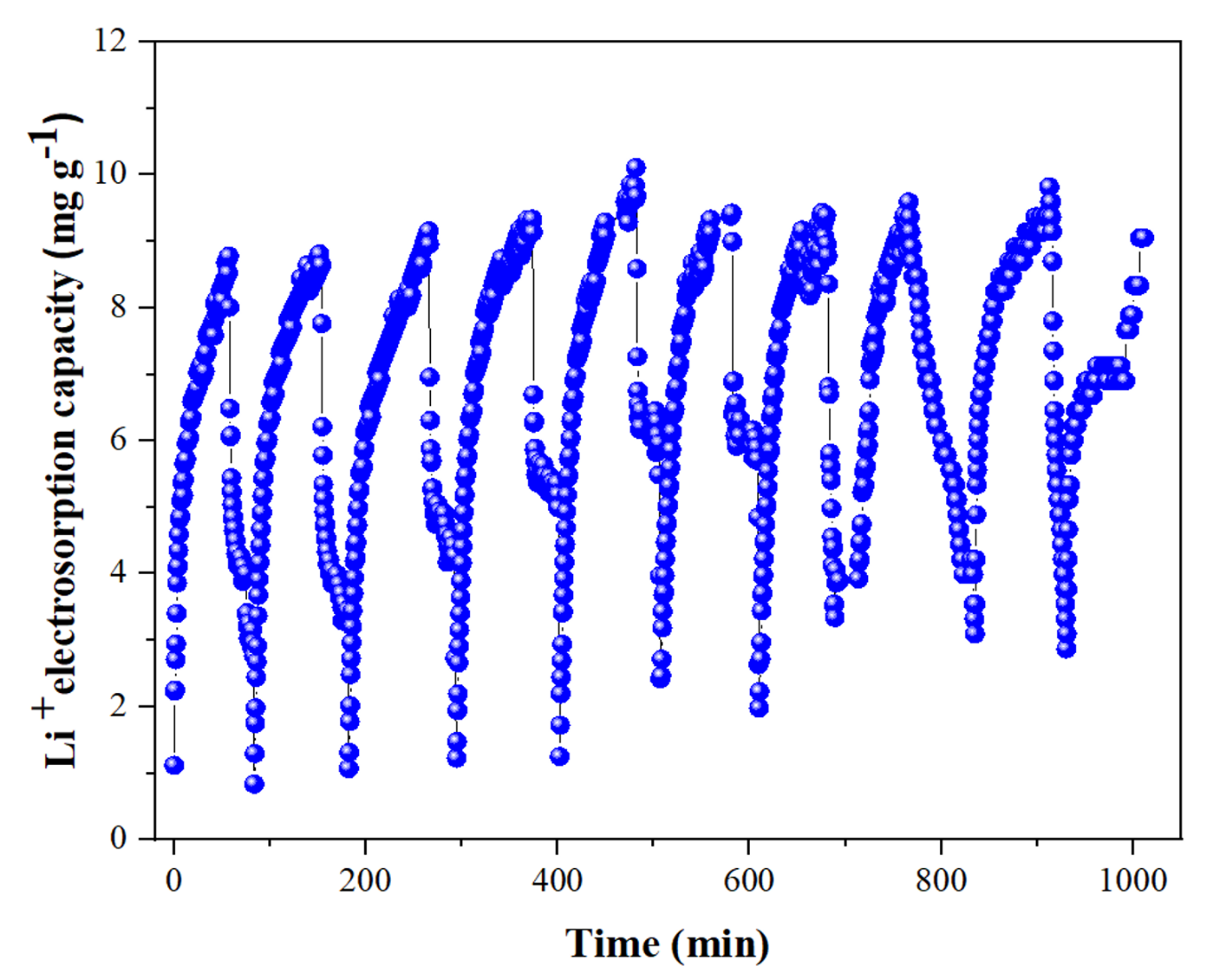


Figure S20: Tta-Dfp electrode cycle stability test with 300 mg L^-1^at 1.2 V


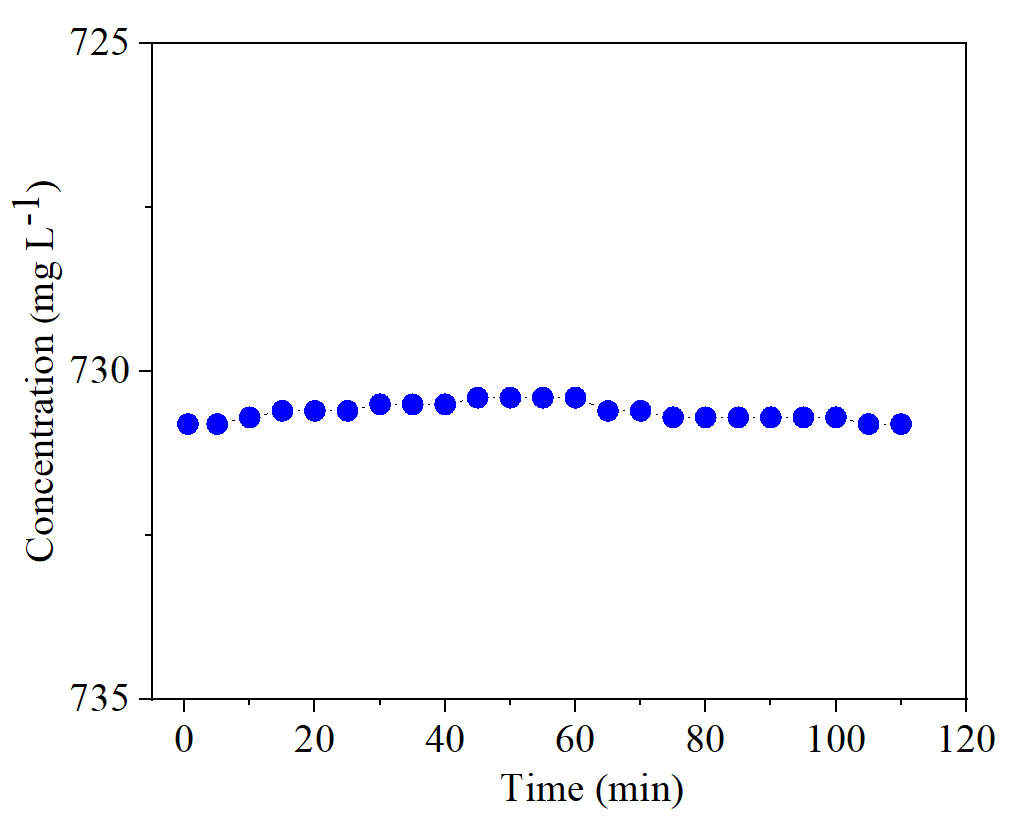


Figure S21: Change of solution concentration for adsorption-desorption experiments of pure graphite in 300 mg L^-1^


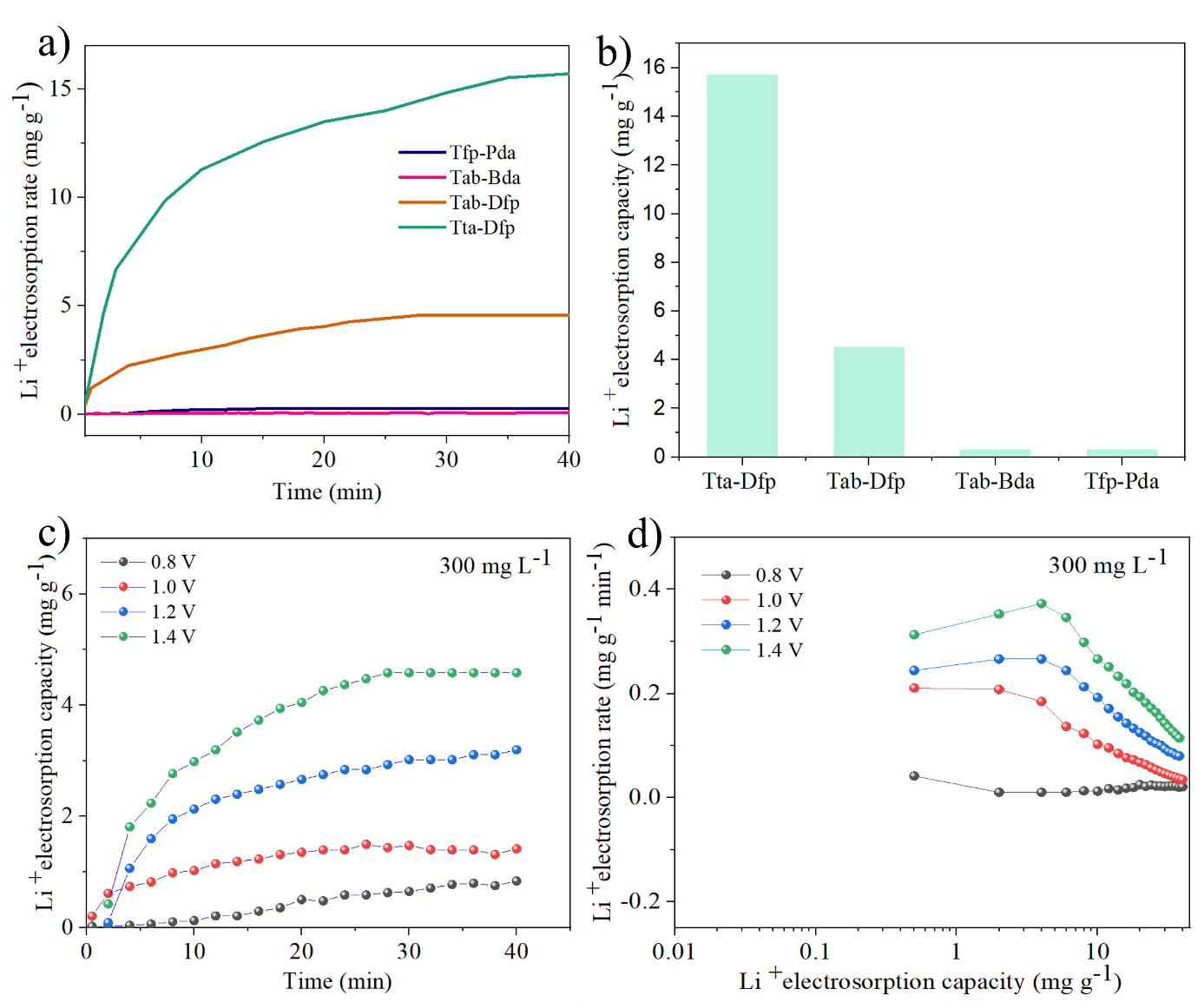


Figure S22: a) The lithium adsorption rate of all COFs; b) The bar diagram of Li+ adsorption capacity Vs COFs with varying nitrogen density; c) the lithium adsorption rate of Tab-Dfp with different voltages; d) Kim–Yoon plot of Tab-Dfp in different voltages in 300 mg L^−1^


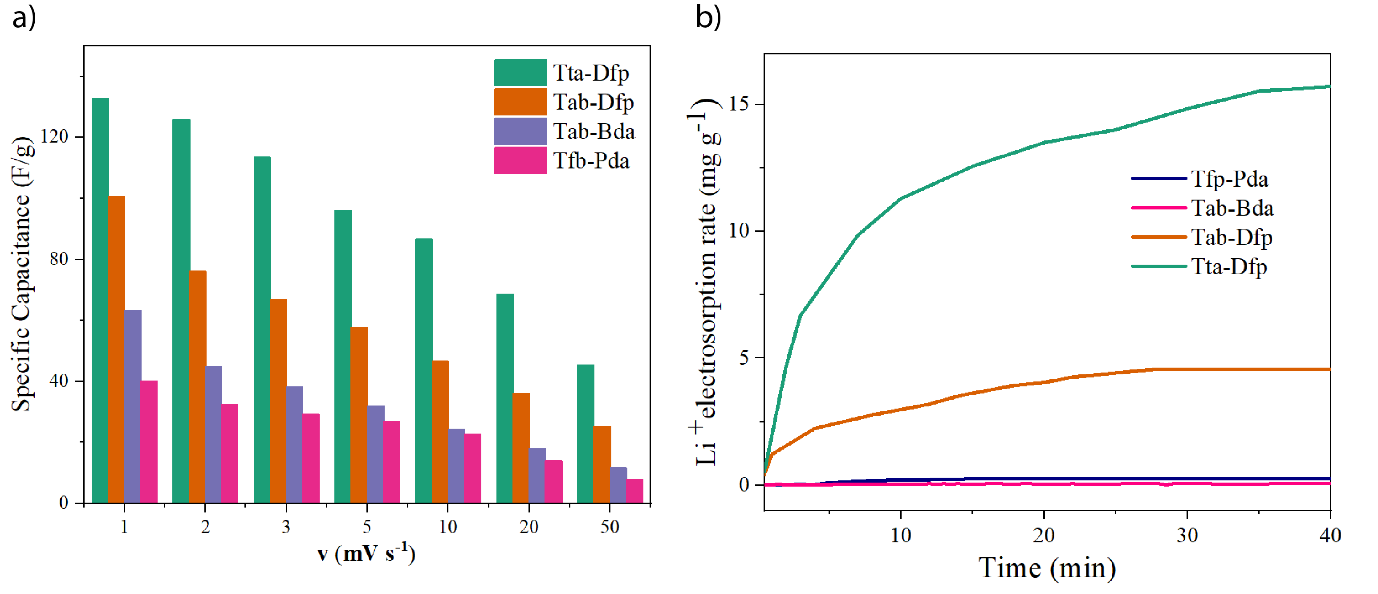


Figure S23: The three-electrode specific capacitance of all COFs


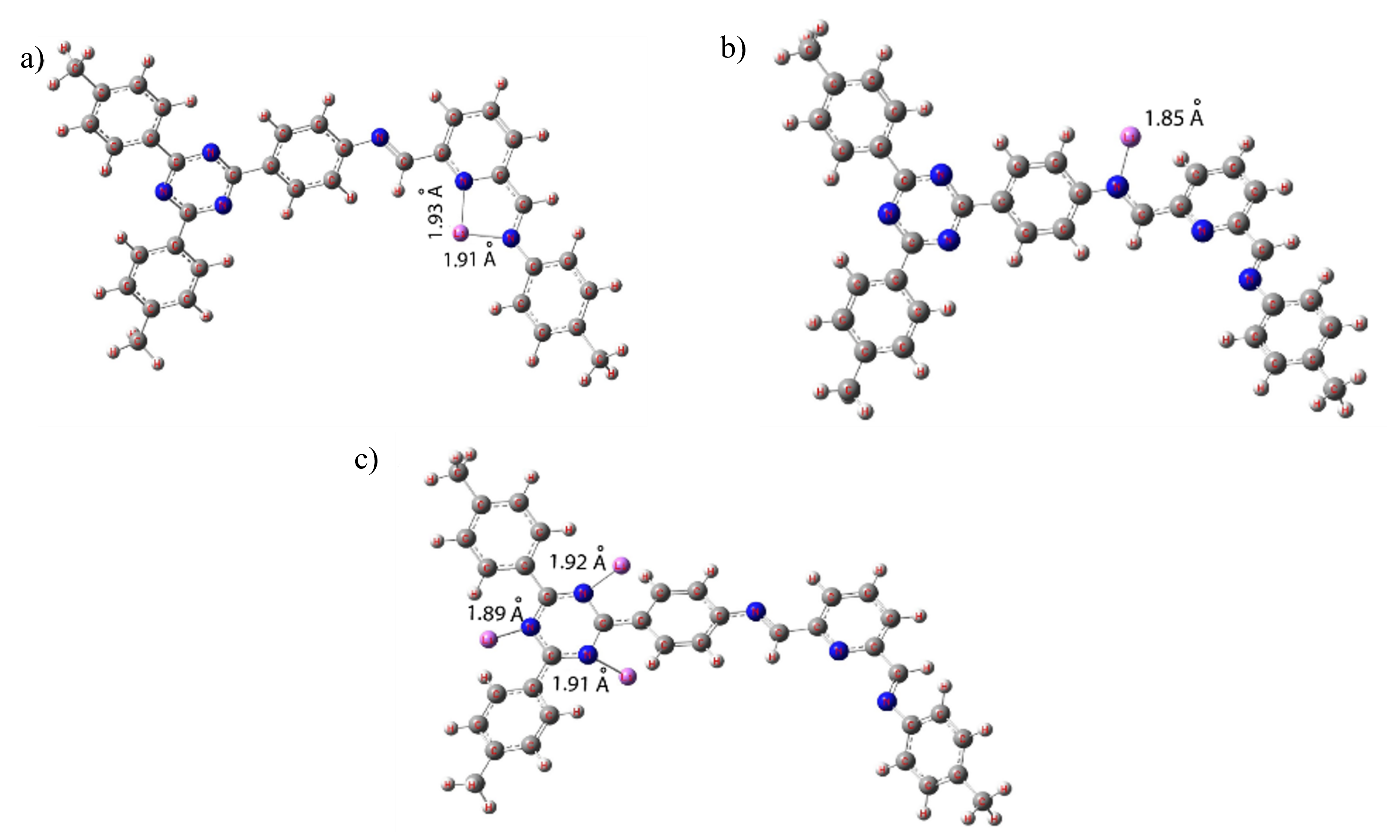


Figure S24: The bond distance and binding energy calculations of lithium interaction with a-c) cis-imine and pyridine nitrogens; trans imine nitrogen and triazine nitrogens.


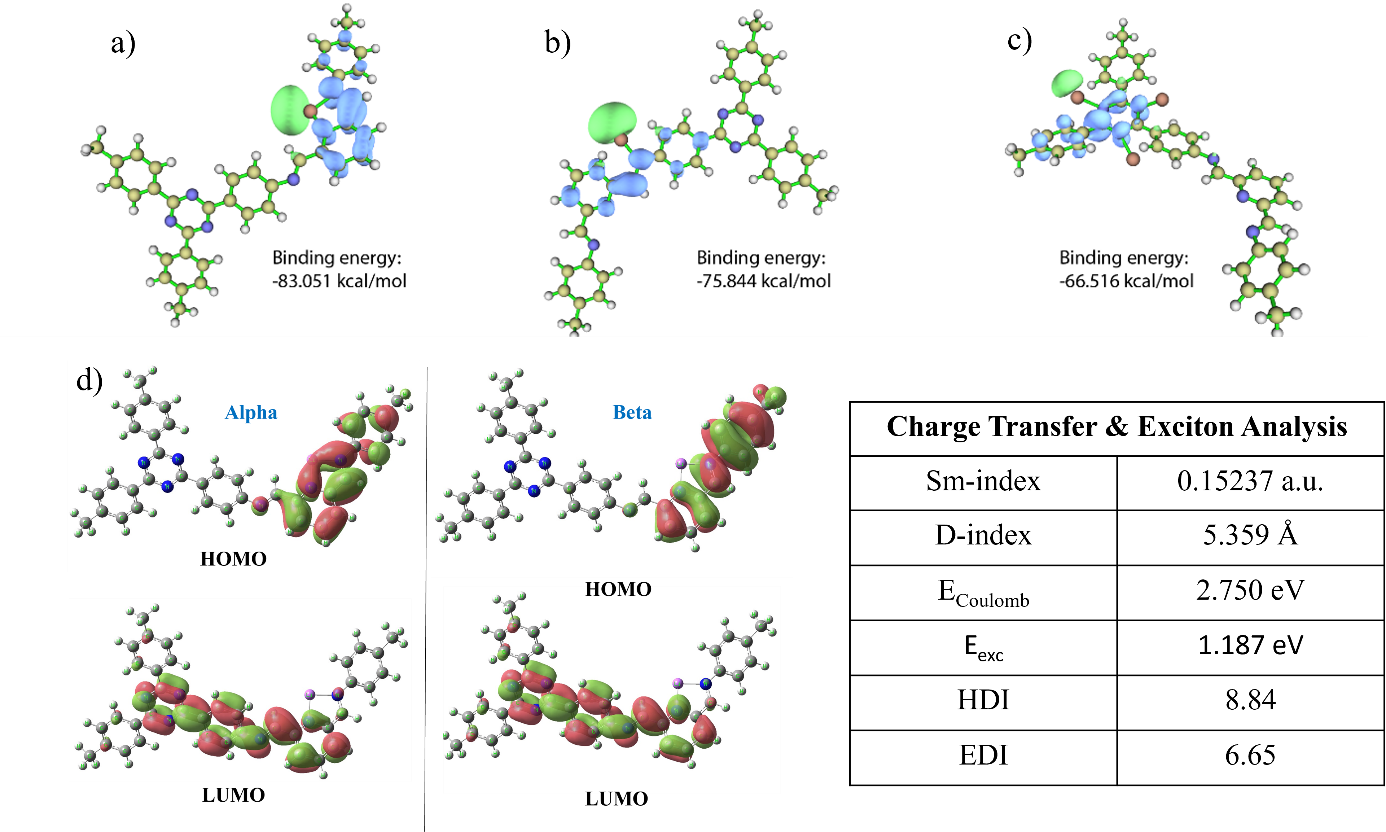


Figure S25: a-c) Charge differential density map (blue-electron accumulation and green hole accumulation) of cis-imine and pyridine nitrogens; trans imine nitrogen and triazine nitrogens. The Binding energy values indicate the strength within the different nitrogen sites of COF, d) Frontier molecular orbitals of Li complex with pyridine and imine nitrogens of Tta-dfp.


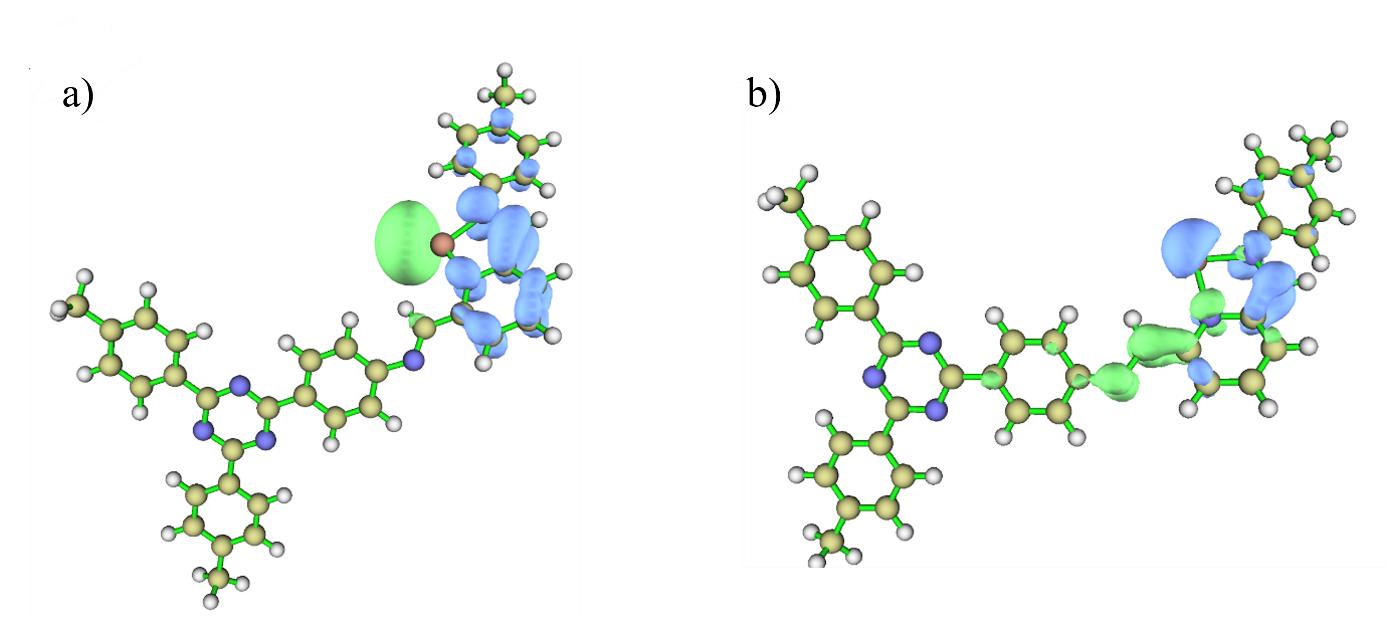


Figure S26: Charge differential densities of Tta-dfp after bonded with a) Li and b) Mg.


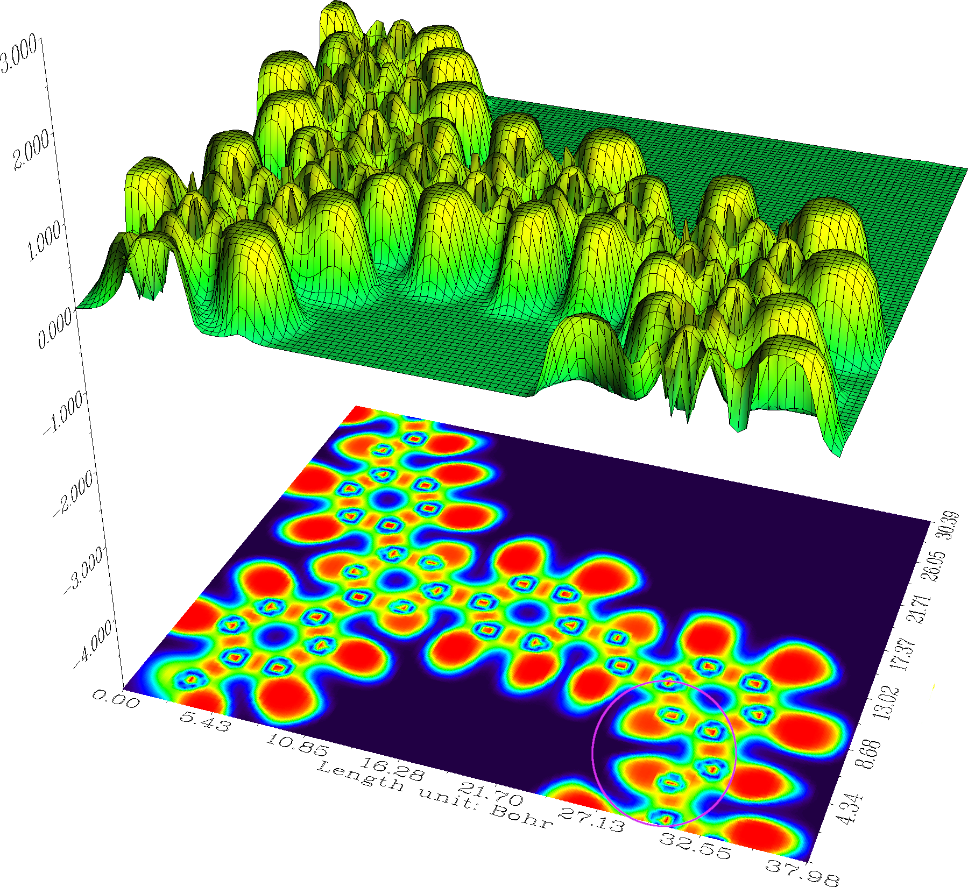


Figure S27: Electron Localization Function (ELF) color filled and contour map of Tta-Dfp repetitive units.


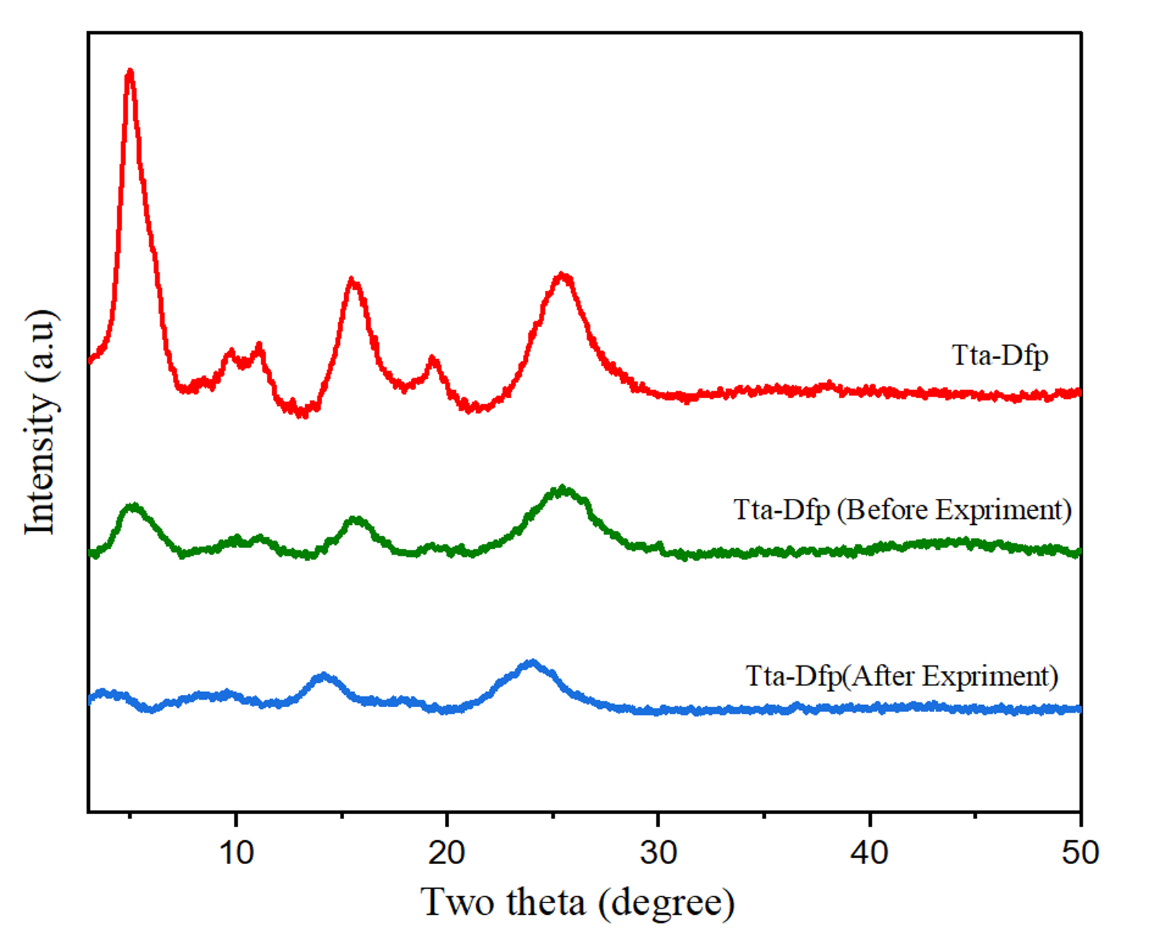


Figure S28: PXRD pattern of Tta-Dfp, Tta-Dfp before and after CDI test


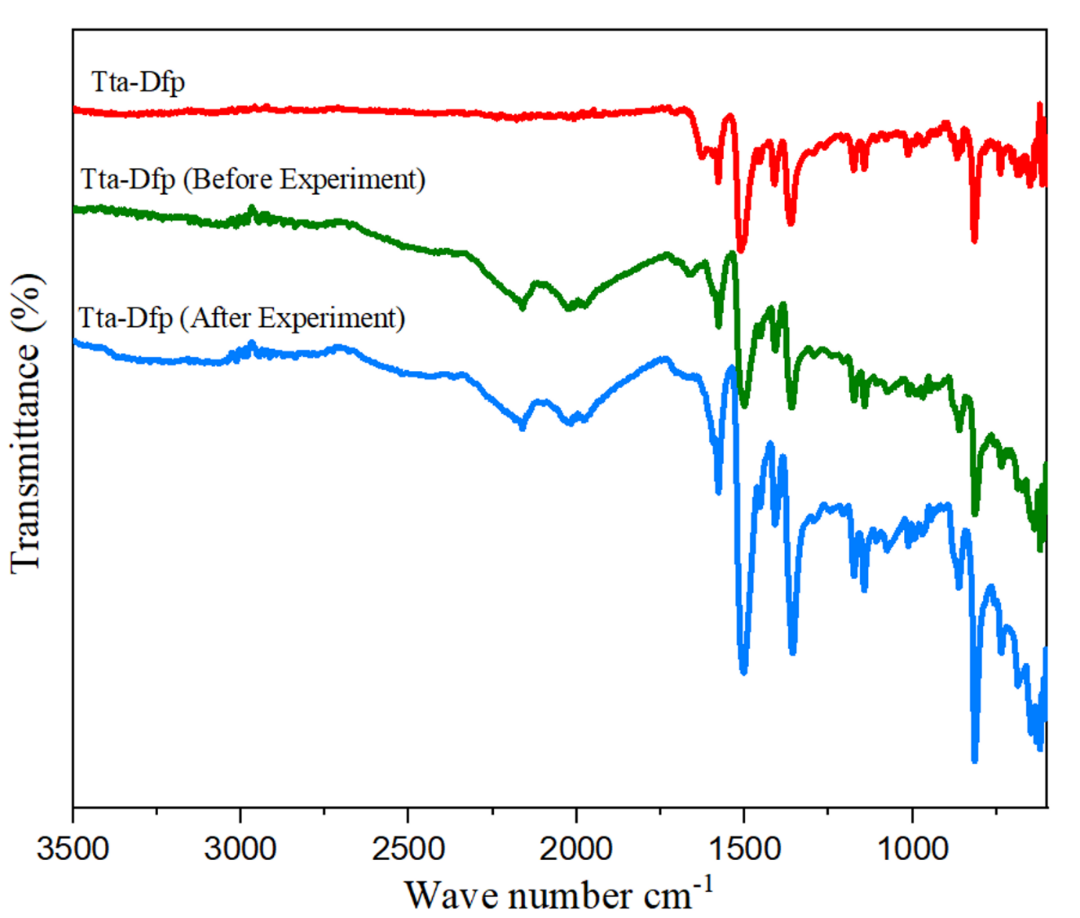


Figure S29: FT-IR analysis of Tta-Dfp, Tta-Dfp before and after CDI test


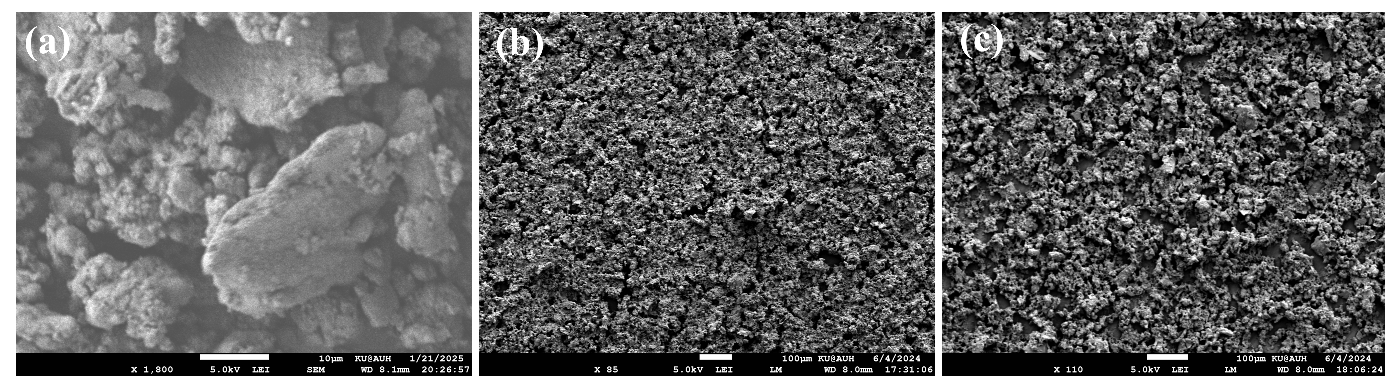


Figure S30: SEM image of a) Tta-Dfp, b) Tta-Dfp of a) Tta-Dfp, b) Tta-Dfp before and (c) after CDI test

Table S1: The R_ct_ values of COFs.

| **COF** | **R_ct_** |
| --- | --- |
| Tta-Dfp | 3.3 |
| Tab-Dfp | 7.4 |
| Tab-Bda | 15.0 |
| Tfp-Bda | 20.8 |

Table S2: Comparison Tta-Dfp electrode and other materials as CDI electrodes.

| **Materials** | **Cell Voltage (V)** | **Concentration (mg L^-1^)** | **Li**^+^ **electrosorption capacity (mg g**^-1^**)** | **CDI system** | **ref** |
| --- | --- | --- | --- | --- | --- |
| SnO_2_/CNT-HC\|\|AC | 1.2 | 500 | 4.00 | HCDI | ^[5]^ |
| LMTO/AC | 2.0 | 424 | 34 | HCDI | ^[6]^ |
| PAN−Li/Al-LDH//AC | - | 100 | 5.2 | HCDI | ^[7]^ |
| Li-TiO_2_-MnO_2_//AC | 1.1 | 423 | 5.89 | MCDI | ^[8]^ |
| LiNi_0.5_Mn_1.5_O_4_/AC | 1.1 | 423 | 11.02 | HCDI | ^[9]^ |
| rGO/NCM\|\|AC | - | 995 | 13.84 | HCDI | ^[10]^ |
| k-MnO_2_/rGO\|\|AC | 1.6 | 423.9 | 25.48 | HCDI | ^[11]^ |
| λ-MnO_2_\|\|AC | 1.0 | 50 | 18.1 | HCDI | ^[12]^ |
| Tta-Dfp//AC | 1.2 | 300 | 10.4 | HCDI | **Present work** |
| Tta-Dfp//AC | 1.4 | 300 | 15.7 | HCDI | **Present work** |

References:

[1] Y. Ha, H. B. Jung, H. Lim, P. S. Jo, H. Yoon, C.-Y. Yoo, T. K. Pham, W. Ahn, Y. Cho, *Energies* **2019**, *12*, 2913.

[2] M. J. Frisch, G. W. Trucks, H. B. Schlegel, G. E. Scuseria, M. A. Robb, J. R. Cheeseman, G. Scalmani, V. Barone, B. Mennucci, G. A. Petersson, H. Nakatsuji, M. Caricato, X. Li, H. P. Hratchian, A. F. Izmaylov, J. Bloino, G. Zheng, J. L. Sonnenberg, M. Hada, M. Ehara, K. Toyota, R. Fukuda, J. Hasegawa, M. Ishida, T. Nakajima, Y. Honda, O. Kitao, H. Nakai, T. Vreven, J. A. Montgomery Jr., J. E. Peralta, F. Ogliaro, M. Bearpark, J. J. Heyd, E. Brothers, K. N. Kudin, V. N. Staroverov, R. Kobayashi, J. Normand, K. Raghavachari, A. Rendell, J. C. Burant, S. S. Iyengar, J. Tomasi, M. Cossi, N. Rega, J. M. Millam, M. Klene, J. E. Knox, J. B. Cross, V. Bakken, C. Adamo, J. Jaramillo, R. Gomperts, R. E. Stratmann, O. Yazyev, A. J. Austin, R. Cammi, C. Pomelli, J. W. Ochterski, R. L. Martin, K. Morokuma, V. G. Zakrzewski, G. A. Voth, P. Salvador, J. J. Dannenberg, S. Dapprich, A. D. Daniels, Ö. Farkas, J. B. Foresman, J. V. Ortiz, J. Cioslowski, D. J. Fox, **n.d.**

[3] C. Lee, W. Yang, R. G. Parr, *Physical review B* **1988**, *37*, 785.

[4] T. Lu, F. Chen, *Journal of computational chemistry* **2012**, *33*, 580–592.

[5] S. Wang, S. Wang, G. Wang, X. Che, D. Li, C. Li, J. Qiu, *Materials Today Communications* **2020**, *23*, 100904.

[6] A. Siekierka, M. Bryjak, *Desalination* **2021**, *520*, 115324.

[7] T. Ding, M. Zheng, Y. Lin, *ACS omega* **2022**, *7*, 11430–11439.

[8] A. Siekierka, J. Kujawa, W. Kujawski, M. Bryjak, *Separation and Purification Technology* **2018**, *194*, 231–238.

[9] X. Shang, B. Hu, P. Nie, W. Shi, T. Hussain, J. Liu, *Separation and Purification Technology* **2021**, *258*, 118009.

[10] X. Zhao, M. Feng, Y. Jiao, Y. Zhang, Y. Wang, Z. Sha, *Desalination* **2020**, *481*, 114360.

[11] B. Hu, X. Shang, P. Nie, B. Zhang, J. Yang, J. Liu, *Journal of Colloid and Interface Science* **2022**, *612*, 392–400.

[12] N. Xie, Y. Li, Y. Yuan, J. Gong, X. Hu, *ACS Applied Energy Materials* **2021**, *4*, 13036–13043.
